# Supplementary material for: Mitochondrial transplantation reduces lower limb ischemia-reperfusion injury by increasing skeletal muscle energy and adipocyte browning
Source: Mol Ther Methods Clin Dev. 2023 Nov 7;31:101152. doi: 10.1016/j.omtm.2023.101152 (PMC10667789; doi:10.1016/j.omtm.2023.101152)
Supplement: Document S1. Figures S1–S13 and Tables S1–S3 [file mmc1.pdf]

OMTM, Volume *31*

Supplemental information

Mitochondrial transplantation reduces lower limb ischemia-reperfusion injury by increasing skeletal muscle energy and adipocyte browning

Jiaqi Zeng, Jianing Liu, Haiya Ni, Ling Zhang, Jun Wang, Yazhou Li, Wentao Jiang, Ziyu Wu, and Min Zhou

Supplementary Information

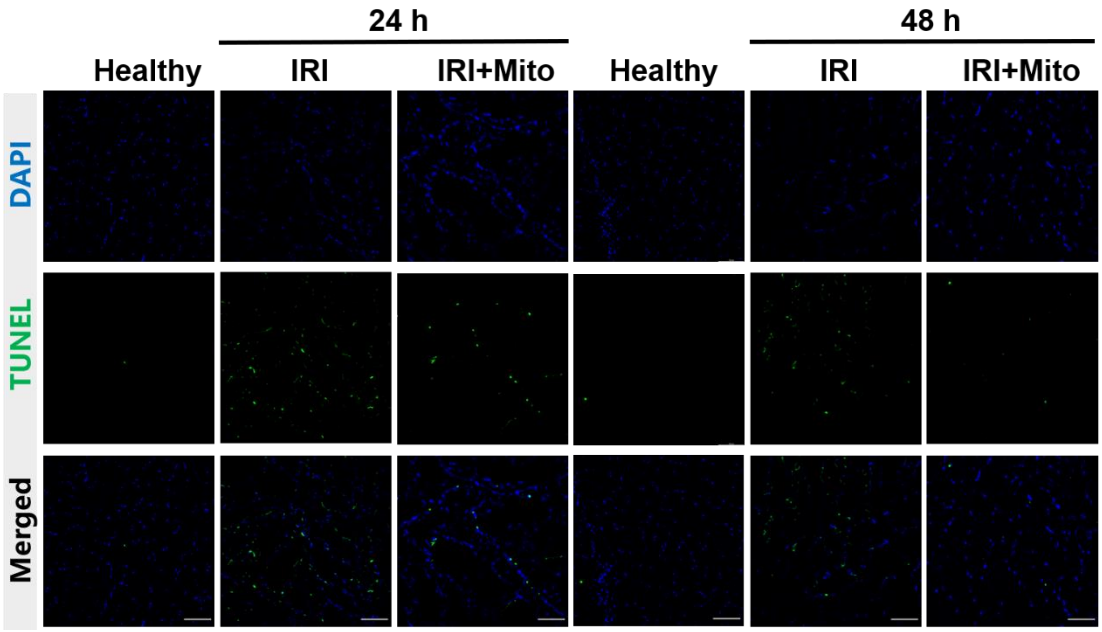

Figure S1. Repair of lower limb IRI by mitochondrial transplantation. Staining of TUNEL-positive cells (blue: nucleus, green: TUNEL, Scale bars: 100 μm).

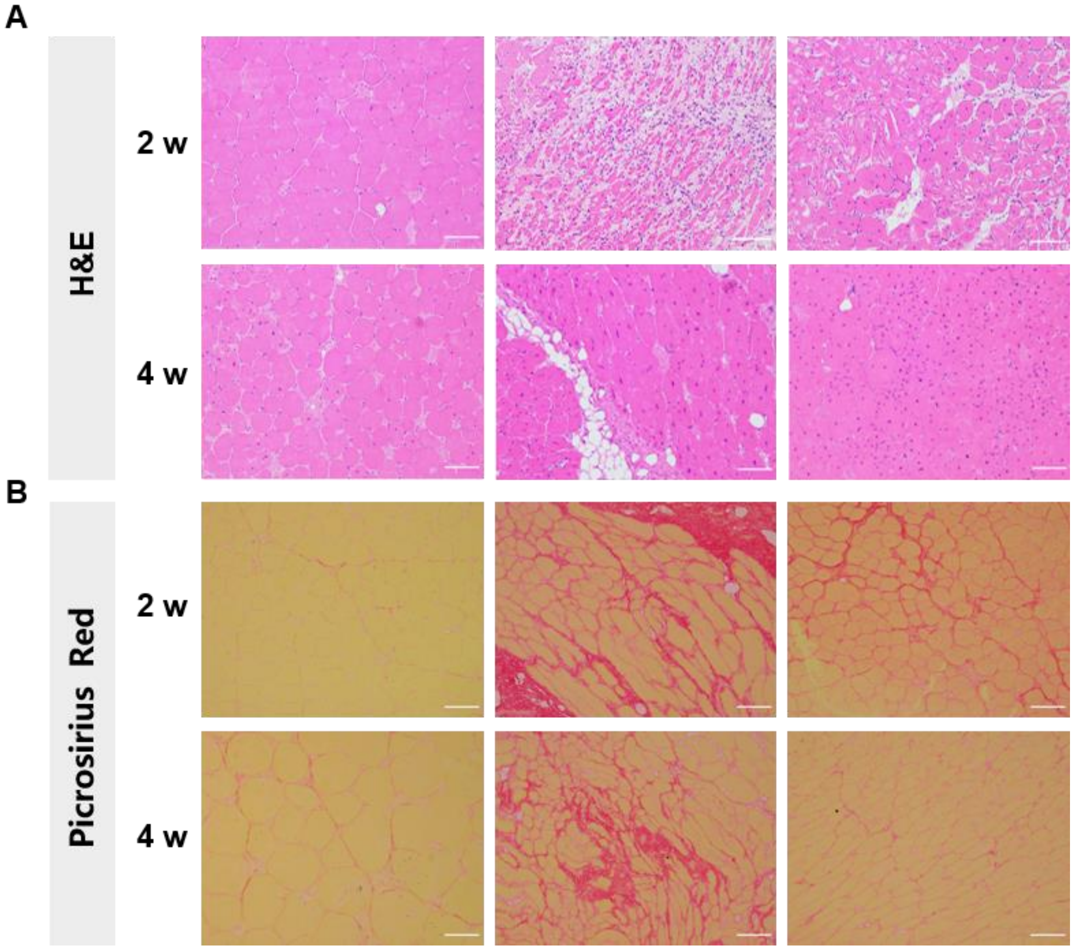

Figure S2. Repair of lower limb IRI by mitochondrial transplantation. (A-B) H&E stain and Picrosirius red stain (Scale bars: 50 μm).

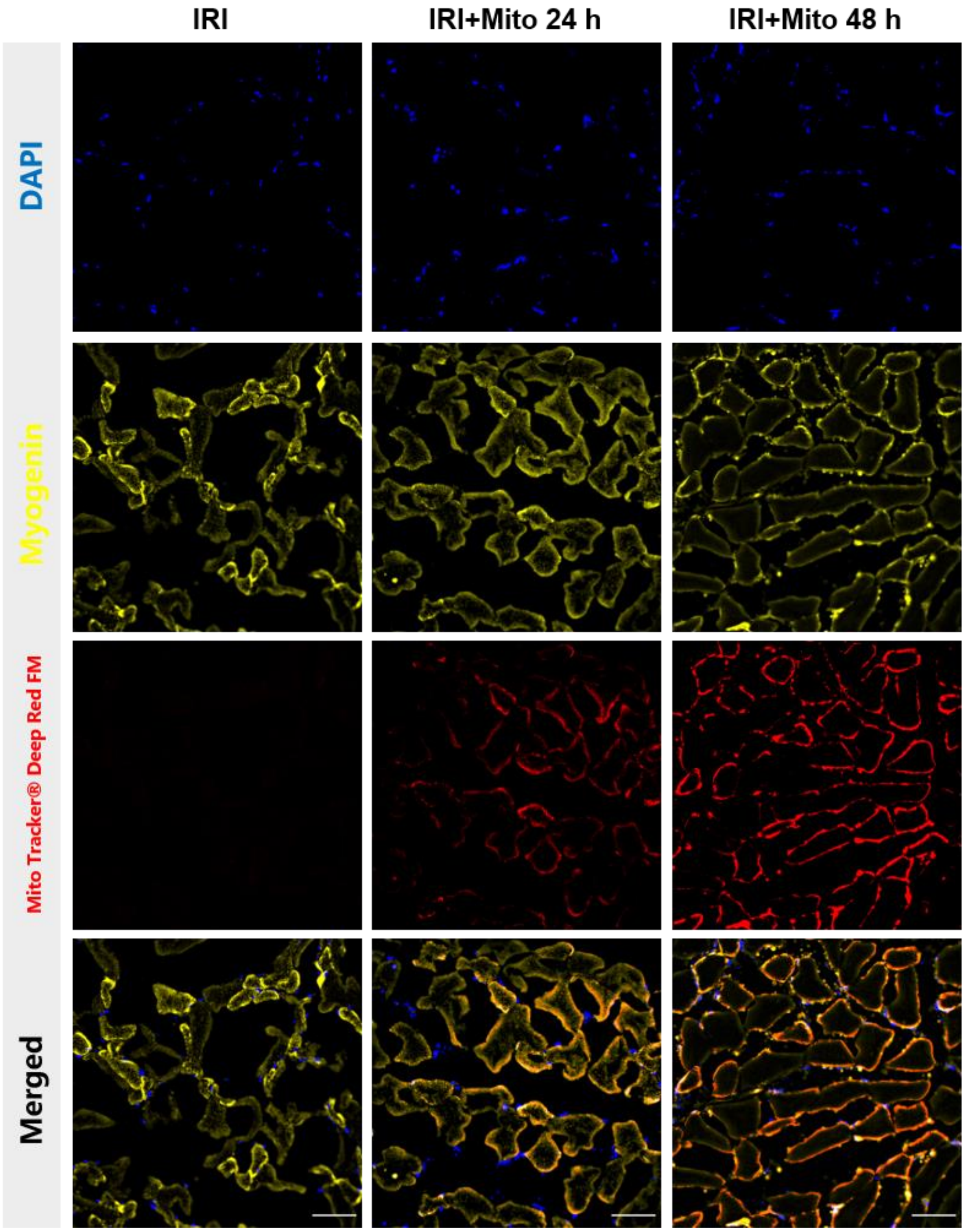

Figure S3. Mitochondrial uptake in *vivo*. CLSM image of hMSCs-derived mitochondria ingestion in mice after 24 h, 48 h of lower limb IRI with mitochondrial transplantation (blue: nucleus, yellow: Myogenin labeled skeletal muscle cells, red: mesenchymal stem cell mitochondria, Scale bars: 50 μm).

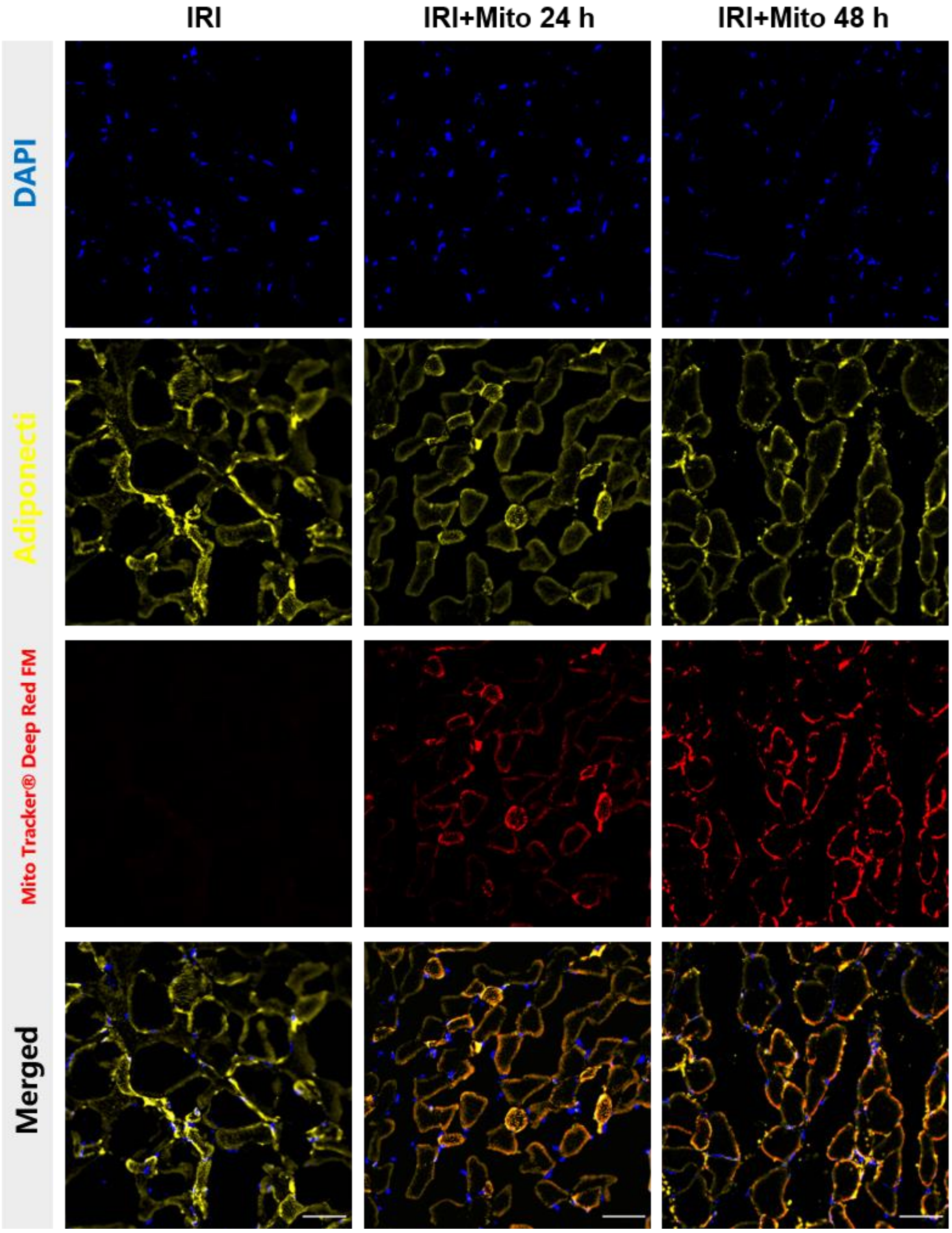

Figure S4. Mitochondrial uptake in *vivo*. CLSM image of hMSCs-derived mitochondria ingestion in mice after 24 h, and 48 h of lower limb IRI with mitochondrial transplantation (blue: nucleus, yellow: adiponectin-labeled adipocytes, red: mesenchymal stem cell mitochondria, Scale bars: 50 μm).

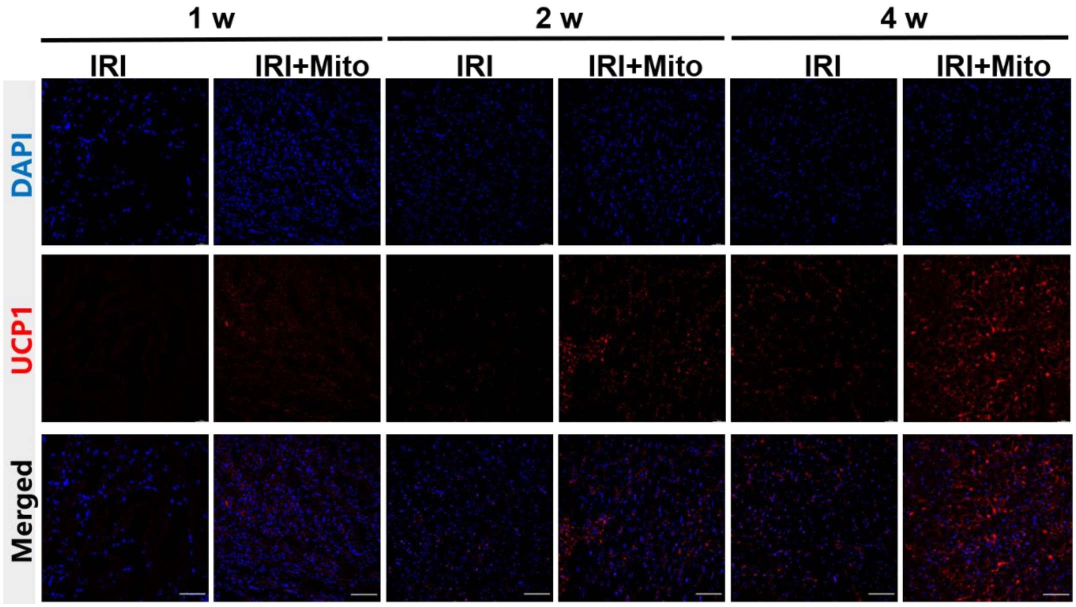

Figure S5. Mitochondrial transplantation promoting effects on the adipocyte browning. Immunofluorescence staining CLSM images was obtained after 1 w, 2 w, and 4 w in the lower limb. (blue: nucleus, red: UCP1, Scale bars: 100 μm)

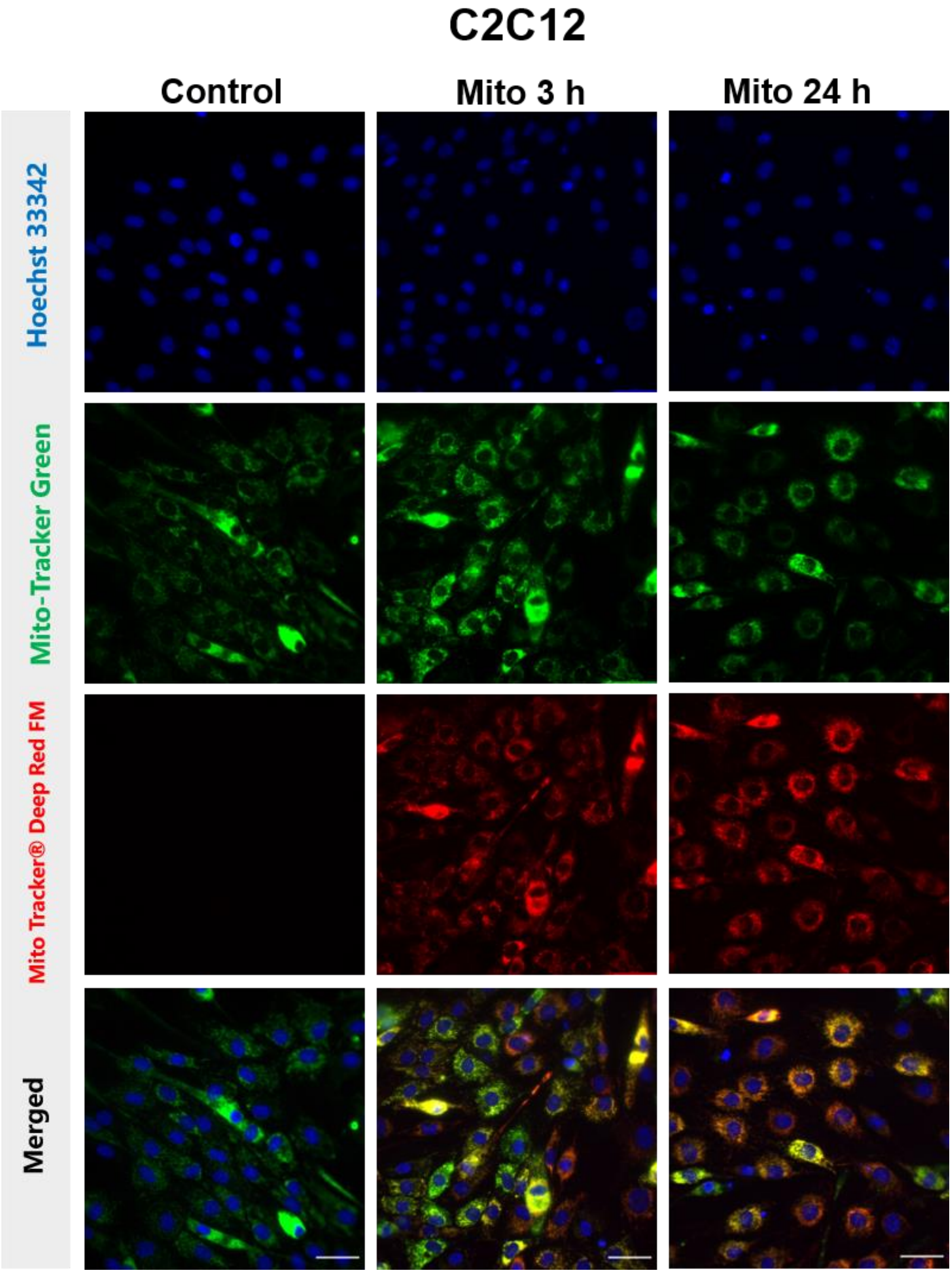

Figure S6. In vitro effects of mitochondrial transplantation on skeletal muscle cells. CLSM images of mitochondrial uptake by C2C12 cells at 3 h and 24 h after hypoxia and reoxygenation. (blue: nucleus, green: original mitochondria of C2C12 cells, red: mitochondria of hMSCs, Scale bars: 50 μm)

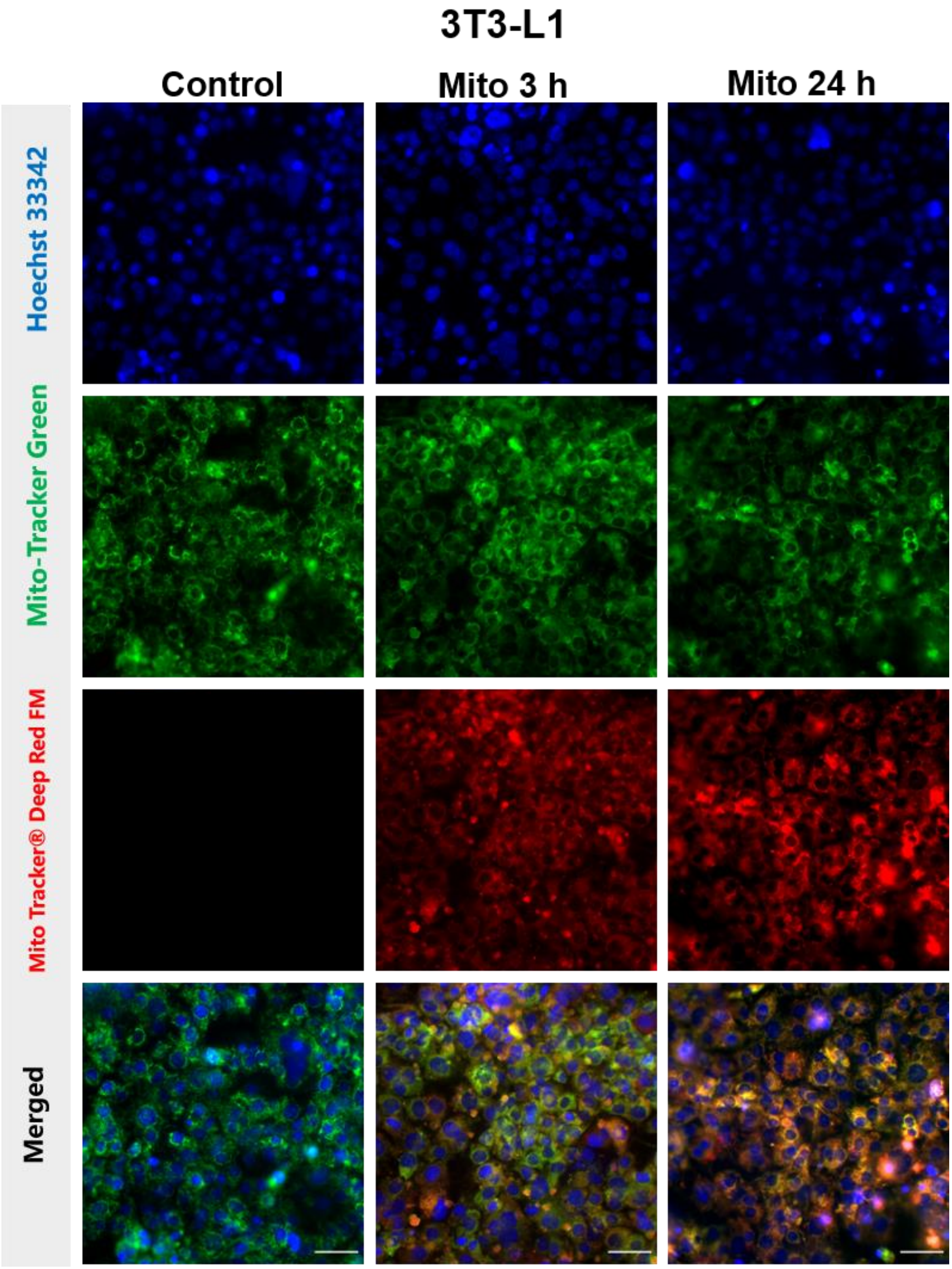

Figure S7. In vitro effects of mitochondrial transplantation on adipocyte browning. CLSM images of mitochondrial uptake by 3T3-L1 cells at 3 h and 24 h. (blue: nucleus, green: 3T3-L1 cell mitochondria, red: hMSCs-derived mitochondria, Scale bars: 50 μm).

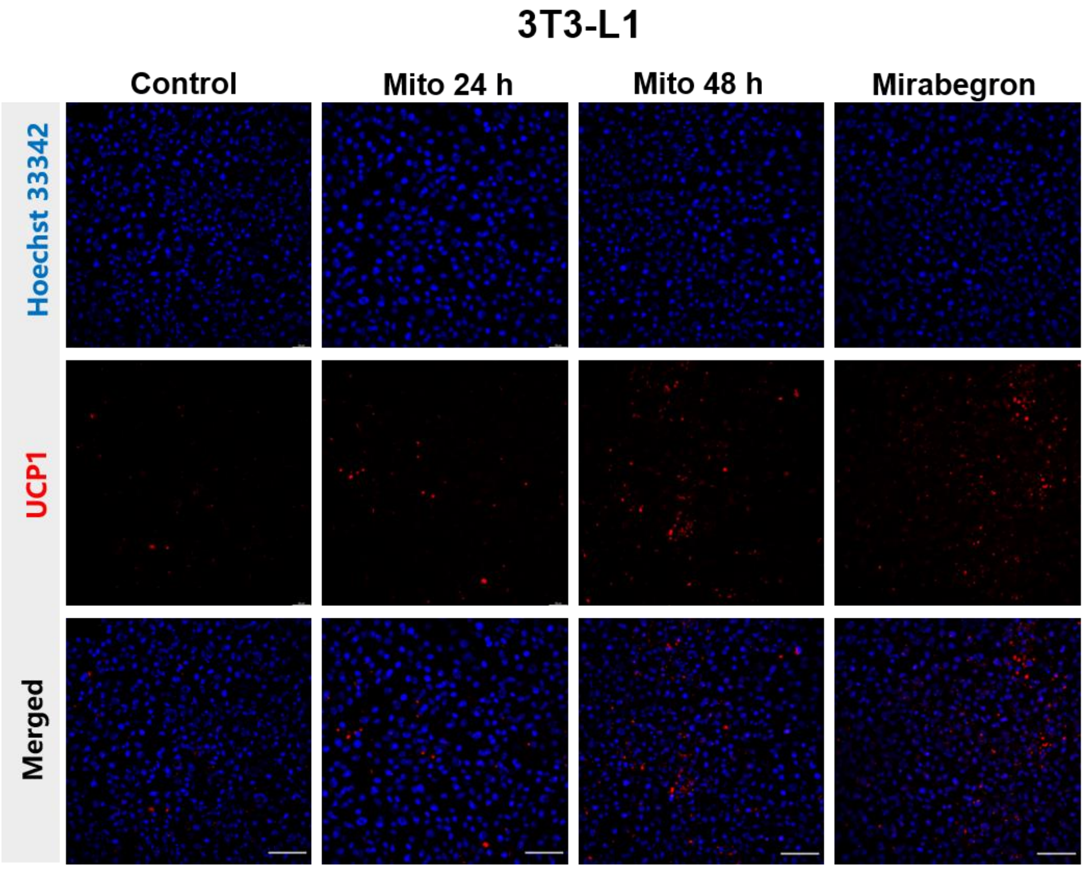

Figure S8. In vitro effects of mitochondrial transplantation on adipocyte browning. CLSM images of UCP1 immunofluorescence. (blue: nucleus, red: UCP1, Scale bars: 100 μm).

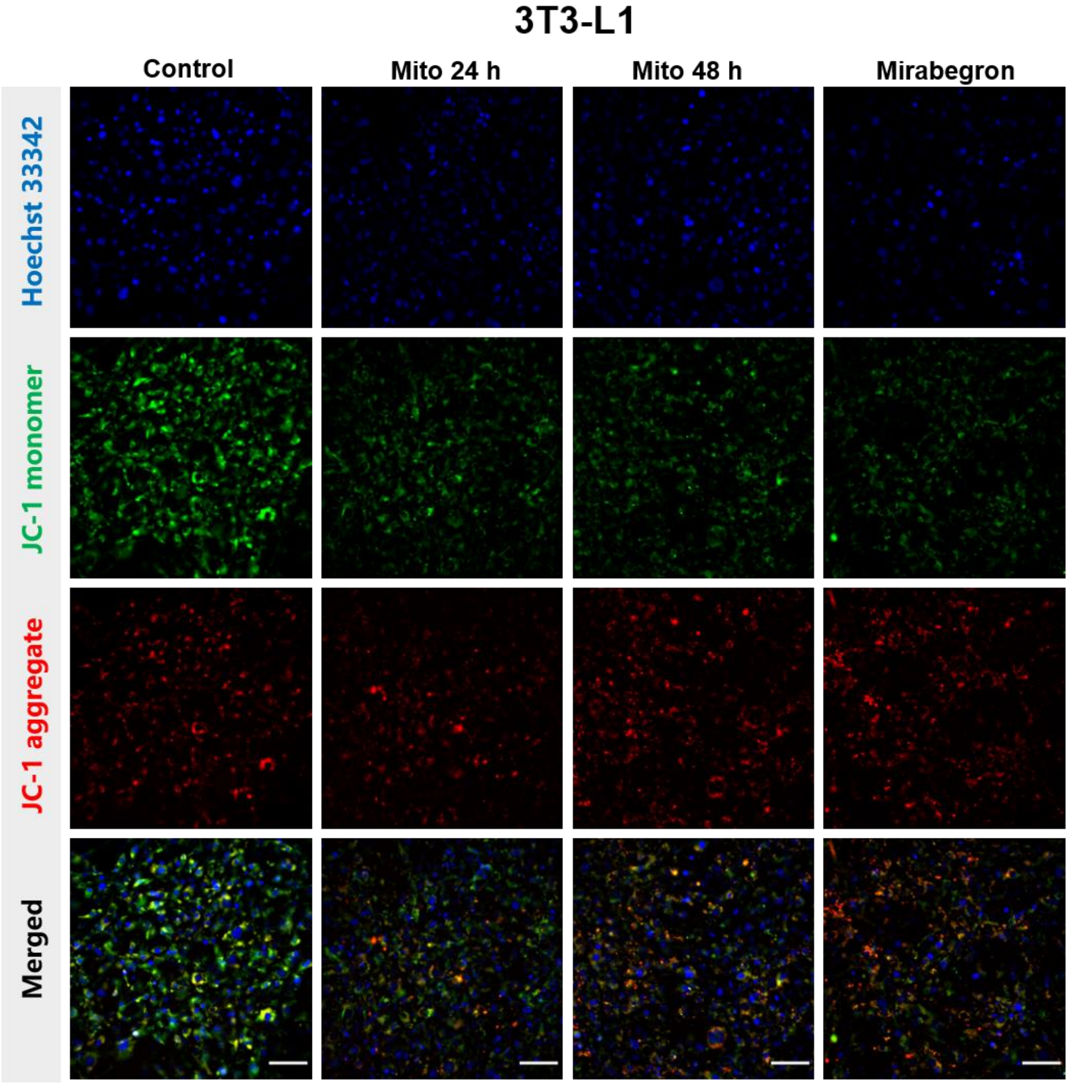

Figure S9. In vitro effects of mitochondrial transplantation on adipocyte browning. JC-1 staining CLSM image (blue: nucleus, green: JC-1 monomer, red: JC-1 aggregate, Scale bars: 100 μm).

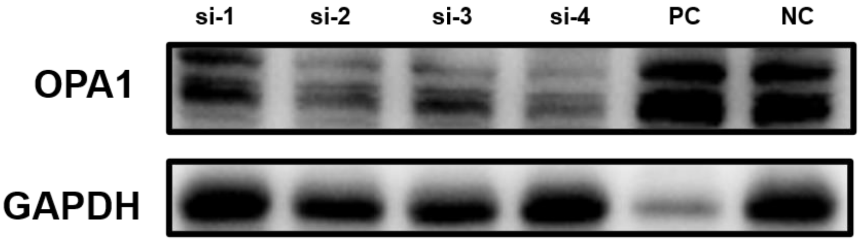

Figure S10. The expression of OPA1 in 3T3-L1-OPA1-siRNA, 3T3-L1-PC-siRNA and 3T3-L1-NC siRNA was compared at the protein level.

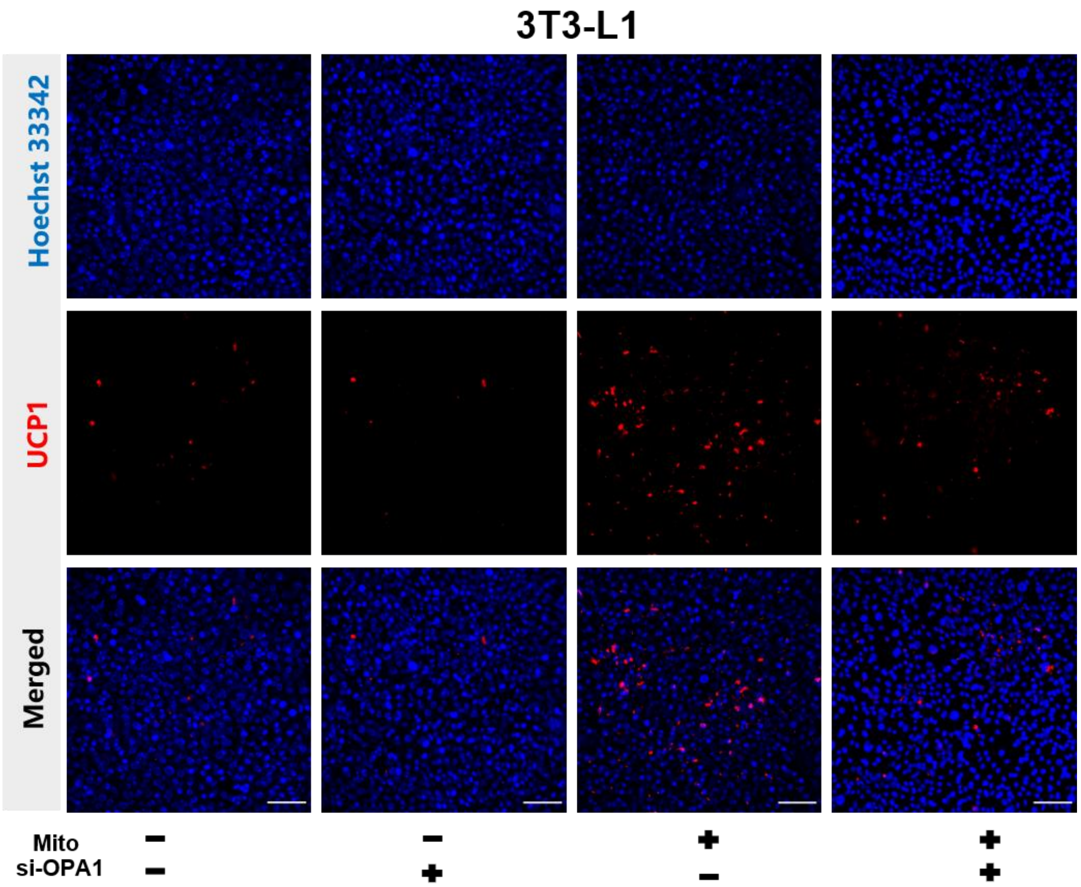

Figure S11. Silencing of OPA1 inhibits adipocyte browning: UCP1 immunofluorescence staining of 3T3-L1 cell blank group, siRNA silencing of OPA1 group, mitochondrial transplantation group, first silencing of OPA1 and then adding mitochondrial group after 48 h of treatment (blue: nucleus, red: UCP1, Scale bars: 100 μm).

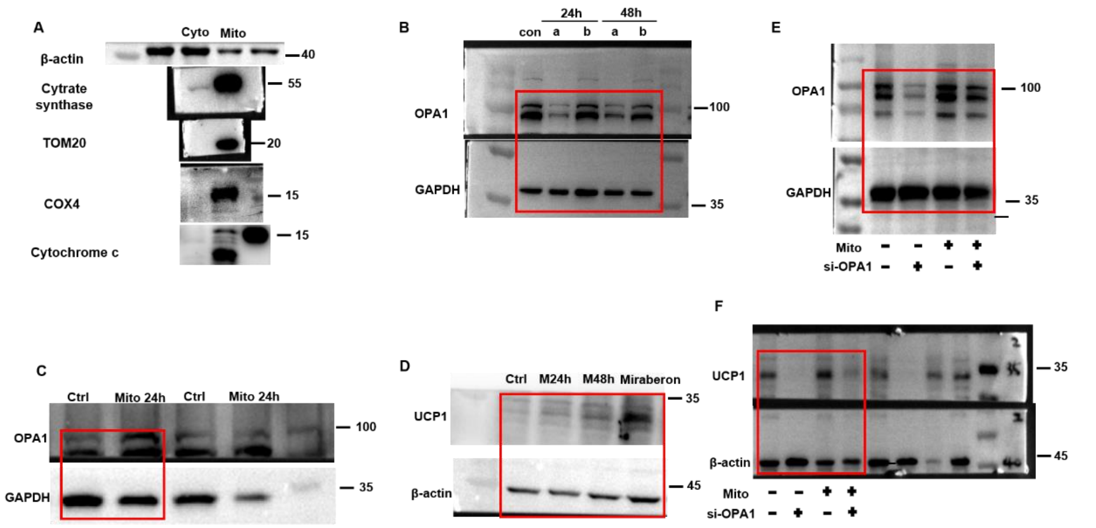

Figure S12. (A) Western blotting analysis of the mitochondria and cytosolic protein markers in the precipitation and supernatant after differential centrifugation. (B) Western blotting of OPA1 in the tissues after 24 h and 48 h (a. lower limb IRI, b. lower limb IRI + Mito). (C) OPA1 protein expression after 24 h of mitochondrial transplantation. (D) Western blotting analysis of UCP1 protein expression. (E-F) Protein levels of OPA1 and UCP1 in 3T3-L1 cell blank group, siRNA silencing OPA1 group, mitochondrial transplantation group, first silencing OPA1 and then adding mitochondria group after 48 h of treatment.

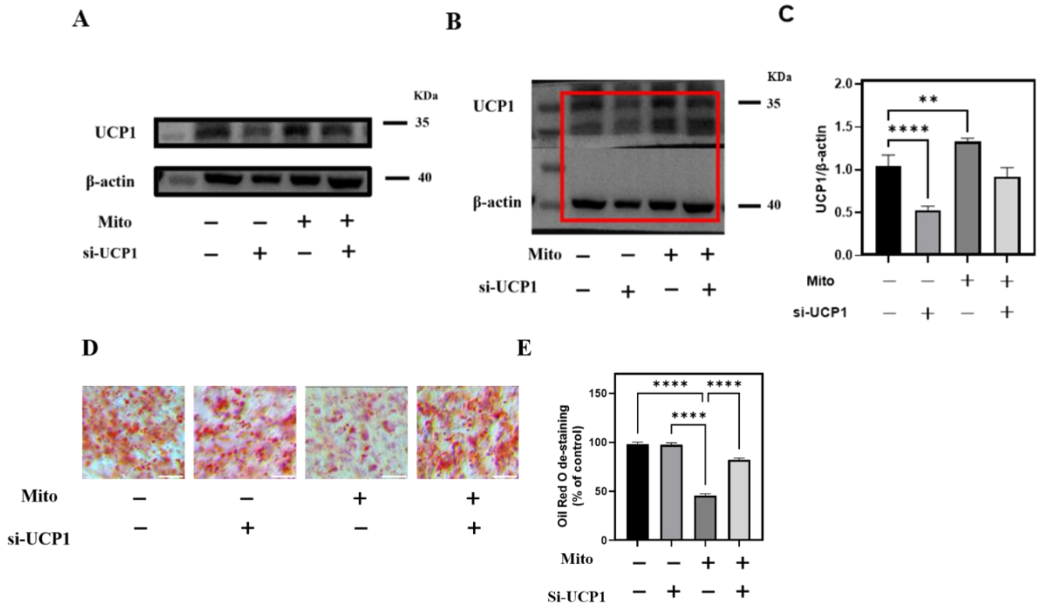

Figure S13. mitochondrial transplantation in the mature adipocytes with knockdown of UCP1. (A-C) The protein expression of UCP1. (D-E) Oil Red O staining and quantification (Scale bar: 2μm). Statistical significance was analyzed using a one-way ANOVA. Experimental data are mean ± s. d. of samples in a representative experiment (n=3). Asterisk (*) denotes statistical significance between bars (**p<0.01, ****

p<0.0001).

Table S1. Results of quantitative analysis of hMSCs mitochondria by Nano-sight

	Stars: Mean+/-Standard Error
**Mean**	337.7+/-23.0 nm
**Mode**	197.3+/-15.4 nm
**SD**	149.4+/-11.2 nm
**D10**	158.4+/-4.4 nm
**D50**	328.6+/-46.3 nm
**D90**	540.9+/-26.4 nm
**Concentration**	5.80e+008+/-1.29e+007particles/ml

Table S2. siRNA2 and siRNA4 (expressed as si-OPA1-2 and si-OPA1-4) sequences used in Western blotting

siRNA name	Forward (5' to 3')	Reverse (5' to 3')
**si-OPA1-2**	CCCGAGACCAUAUCUCUAATT	UUAGAGAUAUGGUCUCGGGTT
**si-OPA1-4**	GUGGCCUUGUUUAAAGAUATT	UAUCUUUAAACAAGGCCACTT

Table S3. si-UCP1 sequences used in Western blotting

siRNA name	Forward (5' to 3')	Reverse (5' to 3')
**si-UCP1**	GCUGUGCGAUGUCCAUGUACATT	UGUACAUGGACAUCGCACAGCTT
