# Supplementary material for: Mitochondrial transplantation reduces lower limb ischemia-reperfusion injury by increasing skeletal muscle energy and adipocyte browning
Source: Mol Ther Methods Clin Dev. 2023 Nov 7;31:101152. doi: 10.1016/j.omtm.2023.101152 (PMC10667789; doi:10.1016/j.omtm.2023.101152)
Supplement: Document S2. Article plus supplemental information [file mmc2.pdf]

Molecular Therapy
Methods & Clinical Development
Original Article

Mitochondrial transplantation reduces lower limb ischemia-reperfusion injury by increasing skeletal muscle energy and adipocyte browning

Jiaqi Zeng,[1,2,4] Jianing Liu,[1,4] Haiya Ni,[1] Ling Zhang,[1] Jun Wang,[1] Yazhou Li,[1] Wentao Jiang,[3] Ziyu Wu,[3] and Min Zhou[1,3]

[1]Department of Vascular Surgery, Nanjing Drum Tower Hospital Clinical College of Nanjing University of Chinese Medicine, Nanjing 210046, China; [2]Department of Vascular Surgery, Kunshan Traditional Chinese Medicine Hospital, Kunshan 215300, China; [3]Department of Vascular Surgery, Nanjing Drum Tower Hospital, The Affiliated Hospital of Nanjing University Medical School, Nanjing 210008, China

Recent studies have shown that mitochondrial transplantation can repair lower limb IRI, but the underlying mechanism of the repair effect remains unclear. In this study, we found that in addition to being taken up by skeletal muscle cells, human umbilical cord mesenchymal stem cells (hMSCs)-derived mitochondria were also taken up by adipocytes, which was accompanied by an increase in optic atrophy 1 (OPA1) and uncoupling protein 1. Transplantation of hMSCs-derived mitochondria could not only supplement the original damaged mitochondrial function of skeletal muscle, but also promote adipocyte browning by increasing the expression of OPA1. In this process, mitochondrial transplantation can reduce cell apoptosis and repair muscle tissue, which promotes the recovery of motor function *in vivo*. To the best of our knowledge, there is no study on the therapeutic mechanism of mitochondrial transplantation from this perspective, which could provide a theoretical basis.

INTRODUCTION

Lower limb ischemia-reperfusion injury (IRI) is a tissue injury caused by blood flow returning to the tissue after ischemia in a short period,[1,2] which often occurs in the process of thrombotic occlusion, embolism, and blood flow recovery after tourniquet use. IRI has become one of the main causes of accidental disability and death after clinical limb revascularization.[3] Lower limb IRI and the mitochondrial dysfunction is considered to be a reciprocal causation, represented by decreased adenosine triphosphate (ATP), calcium overload, increased reactive oxygen species, cell apoptosis, and necrosis.[4–6] These can lead to amyotrophy, myasthenia, and loss of endurance.[7–9] In the process of lower limb IRI, the integrity of mitochondrial function is extremely important for muscle tissue. Current treatment mainly focuses on ischemic pre-conditioning, regional hemofiltration, and controlled reperfusion.[10–14] However, these treatments do not address the mitochondrial damage, the key factor of lower limb IRI.

In recent years, studies have proved that mitochondrial transplantation is an effective treatment for IRI in different organs.[15–18] Some researchers believe that the reason why mitochondrial transplantation is beneficial to repair IRI is that transplanted mitochondria can replace and supplement the original function of damaged mitochondria.[19,20] Among them, it has been proved that stem cell derived-mitochondria can transfer to diseased cells, repairing the lesions, such as heart failure after myocardial infarction, liver IRI, and pulmonary fibrosis.[21–26] However, the long-term effects are not clear. In addition, the underlying therapeutic mechanisms are not thoroughly studied.

Therefore, to explore the therapeutic mechanism of mitochondrial transplantation in lower limb IRI repair, human umbilical cord mesenchymal stem cells (hMSCs)- derived mitochondria of were transplanted to mice with lower limb IRI. We observed mitochondria were taken up by adipocytes in addition to skeletal muscle cells, accompanied by increased expression of optic atrophy 1 (OPA1). Previous studies have shown that elevated OPA1 is conducive to adipocyte browning,[27–29] and adipocyte browning contributes to the recovery of lower limb IRI.[30–32] Therefore, we hypothesized that the transplantation of hMSCs derived-mitochondria may alleviate lower limb IRI by supplementing the original skeletal muscle mitochondrial function as well as promoting adipocyte browning.

RESULTS

Mitochondrial isolation and characterization

To verify that the mitochondria were separated successfully, mitochondria of hMSCs were first marked by Mito Tracker Deep Red

Received 9 June 2023; accepted 3 November 2023;
https://doi.org/10.1016/j.omtm.2023.101152.

[4]These authors contributed equally

Correspondence: Ziyu Wu, Department of Vascular Surgery, Nanjing Drum Tower Hospital, The Affiliated Hospital of Nanjing University Medical School, Nanjing 210008, China.
E-mail: wuziyu@smail.nju.edu.cn
Correspondence: Min Zhou, Nanjing Drum Tower Hospital Clinical College of Nanjing University of Chinese Medicine, Nanjing 210046, China.
E-mail: zhouminnju@nju.edu.cn

© 2023 The Authors.
This is an open access article under the CC BY license (http://creativecommons.org/licenses/by/4.0/).

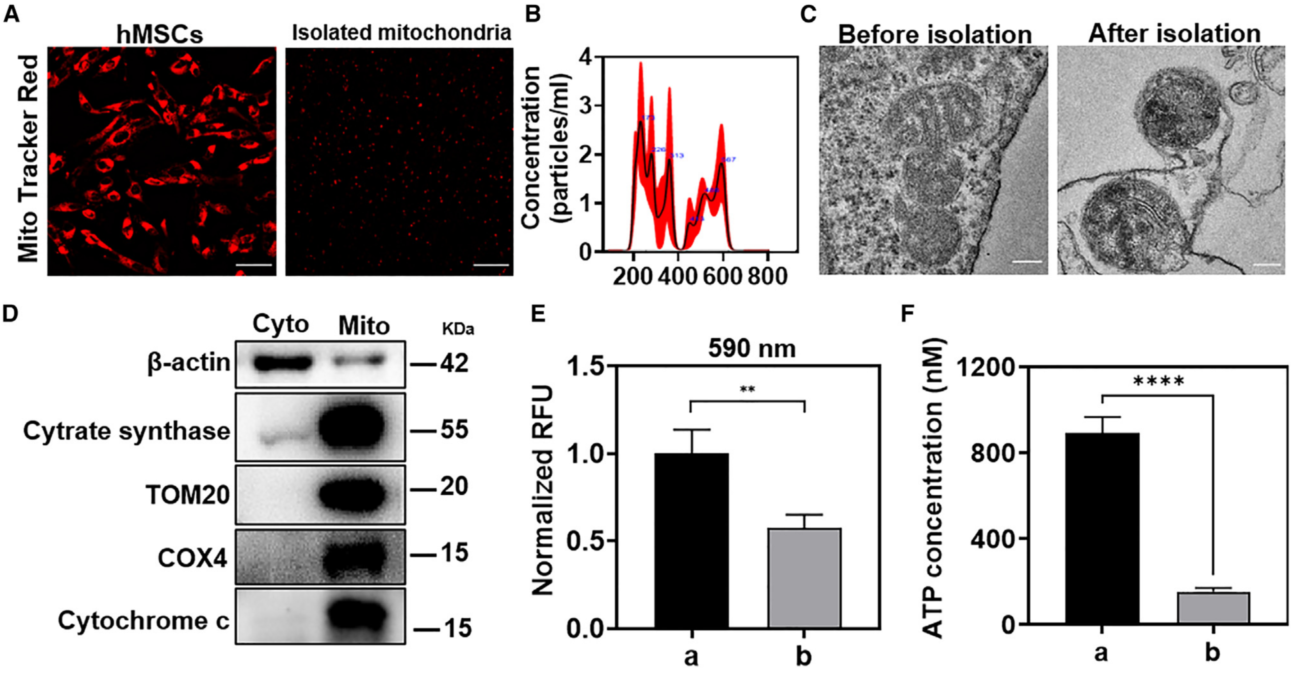

Figure 1. Mitochondrial isolation and characterization

(A) Mito Tracker Deep Red FM-labeled mitochondrial images before and after isolation from hMSCs (scale bars, 50 μm). (B) Nano-Sight results of isolated mitochondria. (C) TEM images of mitochondria before and after isolation from hMSCs (scale bars, 0.2 μm). (D) Western blotting analysis of the mitochondrial and cytosolic protein markers in the precipitation and supernatant after differential centrifugation. (E) Quantitative analysis of JC-1 intensity at 590 nm in (a) Mito and (b) Mito + antimycin A. Statistical significance was analyzed using *t*-tests; (F) Quantitative analysis of ATP in (a) Mito and (b) Mito + antimycin A. Statistical significance was analyzed using Wilcoxon test. Experimental data are mean ± SD of samples in a representative experiment (n = 3). Asterisk (*) denotes statistical significance between bars (**p < 0.01, ****p < 0.0001).

FM probe.[33] The mitochondrial network marked with red fluorescence could be clearly observed in Figure 1A, and the isolated mitochondria also maintained red fluorescence stably after differential centrifugation. The quantitative analysis showed that about 5.8×10^8 mitochondria were isolated from 2×10^7 hMSCs (Figure 1B), and the particle size was distributed at 100–800 nm, with an average of 337.7 nm (Table S1). The integrity of the mitochondrial structural is related to the activity of mitochondria. Therefore, the morphology of the mitochondria was showed by transmission electron microscopy (TEM). Before isolation, the mitochondria in the cells were round and uneven in size. After isolation, the change of the mitochondrial size and morphology were negligible. The mitochondrial ridges were obvious, and the inner and outer membranes were complete (Figure 1C). In addition, the results of western blotting demonstrated the efficacy of differential centrifugation in isolating mitochondria with high purity and integrity. Several mature mitochondrial protein markers were selected to assess isolated mitochondrial function and integrity, including citrate synthase, COX4, cytochrome *c*, and TOM20.[34] Citrate synthase reflects mitochondrial abundance. COX4 reflects mitochondrial functional integrity. Cytochrome *c* reflects the morphological integrity of mitochondria. TOM20 indicates the presence of an intact mitochondrial outer membrane. Higher levels of these mitochondrial markers were observed in the mitochondrial fraction compared with the cytoplasmic fraction, as shown in Figures 1D and S12A. In contrast, the lower presence of the cytosolic protein marker β-actin in the mitochondrial fraction supported the successful separation of mitochondria from the cytoplasm. By using these widely recognized mitochondrial markers, we are able to confirm the integrity and purity of isolated mitochondria, which provides confidence for subsequent analyzes and mitochondrial transplantation experiment. Next, we demonstrated the activity of mitochondria by measuring mitochondrial membrane potential and ATP synthesis capacity. As shown in Figure 1E, the treatment of antimycin A (inhibitor of mitochondrial respiration) (i.e., mitochondria were treated with 1 mM antimycin A for 30 min) reduces the fluorescence intensity of JC-1 probe at 590 nm. This can be attributed to the inhibition of mitochondrial respiration by antimycin A, which leads to the collapse of mitochondrial membrane potential and the decrease of the JC-1 aggregate.[35] Therefore, the isolated mitochondria were proved to be reactive to antimycin A. In terms of ATP synthesis, the isolated mitochondria were more efficient in ATP synthesis than the mitochondria treated with antimycin A (i.e., mitochondria were treated with 1 mM antimycin A for 30 min) when providing substrates malate, glutamate, and ADP (Figure 1F).[36]

Mitochondrial transplantation repair lower limb IRI

Mitochondrial transplantation had been shown to promote the repair of lower limb IRI.[37] We established a model of lower limb IRI in mice

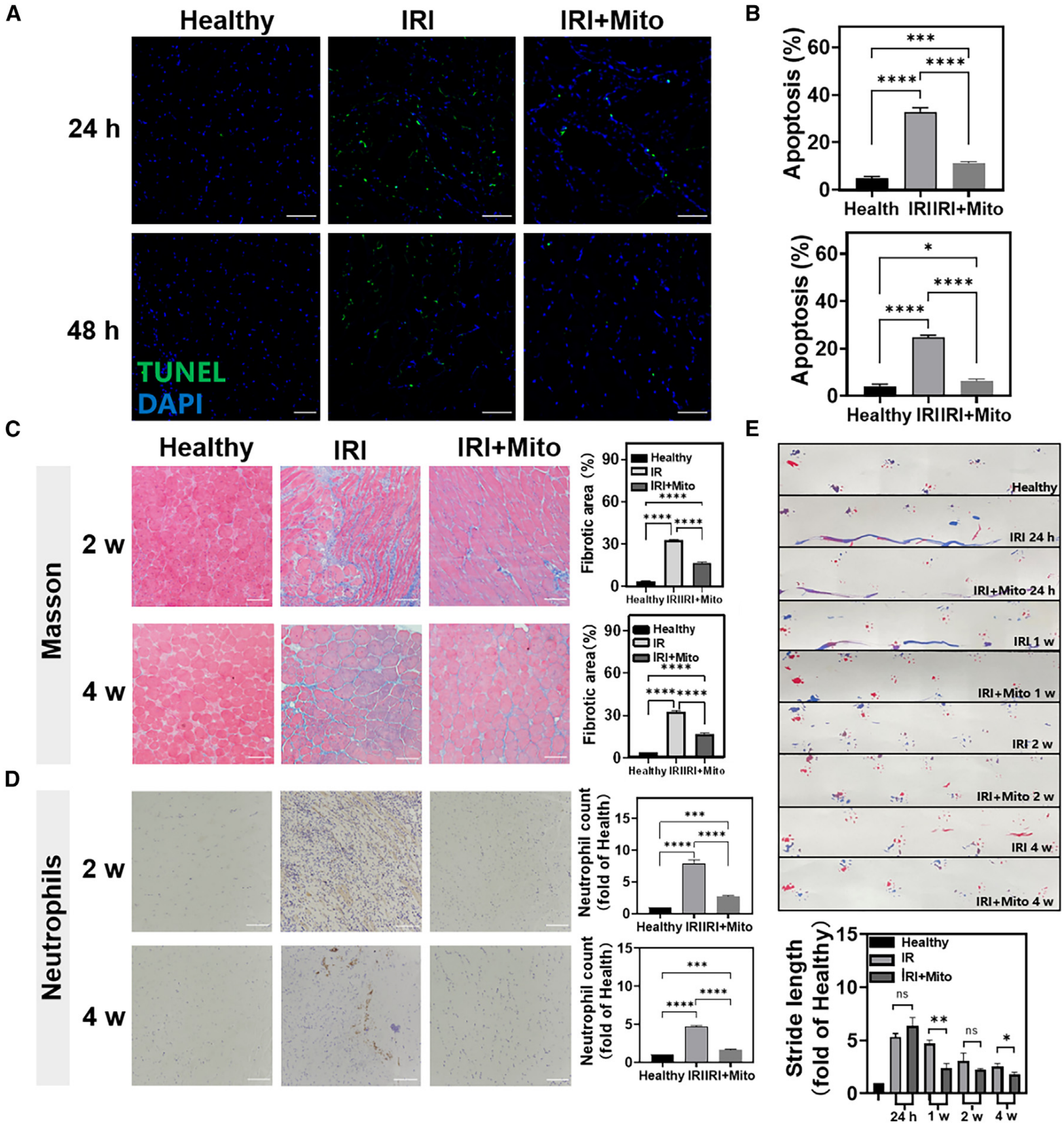

Figure 2. Repair of lower limb IRI by mitochondrial transplantation

(A and B) Staining of TUNEL-positive cells (blue, nucleus; green, TUNEL; scale bars, 100 μm). (C) Masson staining of muscle tissue at 2 weeks and 4 weeks (scale bars, 50 μm). (D) Neutrophils staining of muscle tissue at 2 weeks and 4 weeks (scale bars, 50 μm). Statistical significance was analyzed using a one-way ANOVA. (E) Footprint images. Red is the forelimb footprint, blue is the hindlimb footprint. Statistical significance was analyzed using Kruskal-Wallis test. Experimental data are mean ± SD of samples in a representative experiment (n = 3). Asterisk (*) denotes statistical significance between bars (*$p < 0.05$, **$p < 0.01$, ***$p < 0.001$, ****$p < 0.0001$).

by ligating the right lower limb with rubber bands, and the isolated mitochondria were injected into the gastrocnemius muscle after 15 min of restoring perfusion. The healthy side limb served as a control. In the early stage of treatment, the apoptosis was assessed by TUNEL staining. Compared with healthy tissues, there were more apoptotic cells in lower limb IRI tissues after 24 h and 48 h of treatment, while the mitochondrial transplantation reduced cell apoptosis (Figures 2A, 2B, and S1). The healthy mice were taken as controls. To verify the long-term effect of mitochondrial transplantation, we evaluated the morphology of the tissues after 2 weeks and 4 weeks of the treatment. Masson staining and neutrophils staining (Figures 2C and 2D) showed that after 2 weeks of the treatment, the inflammatory cells infiltration and tissue exudation of the mitochondrial transplantation group were reduced compared with the healthy group. After 4 weeks of the treatment, the arrangement of muscle fiber was tight, and the nucleus was on the edge of the muscle fiber. On the whole, the

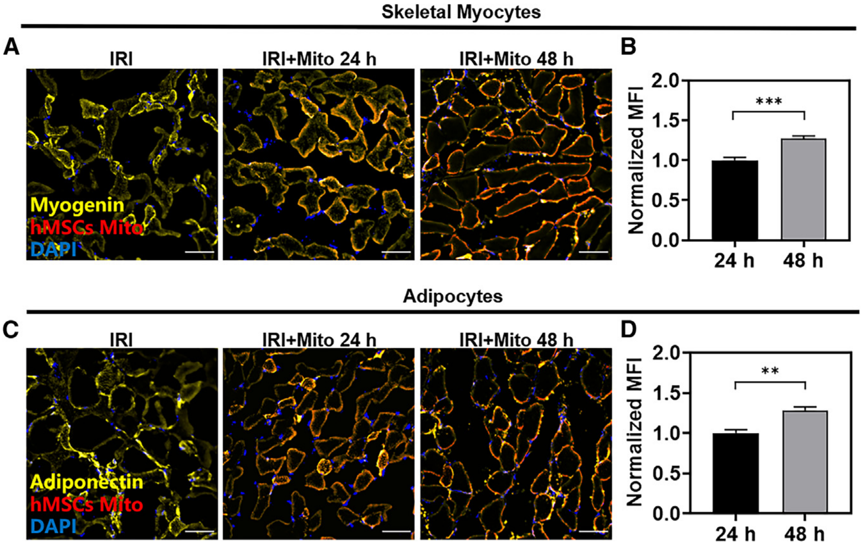

Figure 3. Mitochondrial uptake in vivo

(A) CLSM image of hMSCs-derived mitochondria ingestion in mice after 24 h and 48 h of lower limb IRI with mitochondrial transplantation (blue, nucleus; yellow, Myogenin labeled skeletal muscle cells; red, mesenchymal stem cell mitochondria; scale bars, 50 μm). (B) Changes in the mean fluorescence intensity of hMSCs-derived mitochondria ingested by skeletal muscle cells. Statistical significance was analyzed using *t*-tests. (C) CLSM image of hMSCs-derived mitochondria ingestion in mice after 24 h and 48 h of lower limb IRI with mitochondrial transplantation (blue, nucleus; yellow, adiponectin-labeled adipocytes; red, mesenchymal stem cell mitochondria; scale bars, 50 μm). (D) Changes in the mean fluorescence intensity of hMSCs-derived mitochondria ingested by adipocytes. Statistical significance was analyzed using *t*-tests. Experimental data are mean ± SD of samples in a representative experiment (n = 3). Asterisk (*) denotes statistical significance between bars (**$p < 0.01$, ***$p < 0.001$).

form of muscle fiber was restored to normal, and the skeletal muscle had almost completely regenerated.[38] At the same time, hematoxylin and eosin staining and Picrosirius Red staining (Figure S2) showed that the collagen fibers of the injured muscle tissue in the mitochondrial transplantation group were significantly reduced compared with the lower limb IRI group. With the increase of time, especially in the fourth week, the collagen fibers in the mitochondrial transplantation group had almost no deposits, and the degree of fibrosis was significantly alleviated. To further evaluate the effects of mitochondrial transplantation on functional recovery of the lower limb, the footprint assessment was performed at 24 h, 1 week, 2 weeks, and 4 weeks (Figure 2E). The forelimb footprints were displayed in red and the hindlimb footprints is blue. The recovery of hind limbs can be reflected in the length of stride, the overlap of the front paw and rear claws, and the traces of drag. It showed that the gait of mice in the mitochondrial transplantation group was significantly better than that in the lower limb IRI group. In conclusion, mitochondrial transplantation had significantly repaired lower limb IRI.

Mitochondrial uptake *in vivo*

Some studies have suggested that the transplanted mitochondria play a therapeutic role by supplementing the mitochondrial function of receptor cells.[19] As the main cell subpopulation of lower limb muscle, skeletal muscle cells may be the main receptor cells of transplanted mitochondria. However, the possibility of the transplanted mitochondria uptake by other cell groups cannot be ruled out because the reconstruction of lower limb tissue involves multiple cell types.[39] Therefore, we first studied the uptake of transplanted mitochondria by different cells in the model of lower limb IRI. As expected, the mitochondria marked with red fluorescence colocalized with the skeletal muscle cells marked with Myogenin after 24 h of the intramuscular injection (Figures 3A, 3B, and S3). Interestingly, we also found that these mitochondria colocalized with Adiponectin-labeled adipocytes (Figures 3C, 3D, and S4). This suggested that adipocytes might take mitochondria up. In addition, the intensity of red fluorescence in both skeletal muscle cells and adipocytes gradually increased, indicating that the uptake of the mitochondria had gradually increased.

Mitochondrial transplantation promoting effects on the adipocyte browning

Adipose tissue had been shown to play an important role in the progression of lower limb ischemic disease. The brown adipose tissue had highly metabolic activity, which is conducive to the repair of lower limb ischemia.[32] Based on the fact that the process of adipocyte browning is closely related to mitochondria and the results of mitochondrial uptake by adipocytes mentioned above,[40] we studied the effect of mitochondrial transplantation on adipocyte browning in the model of lower limb IRI. The adipocyte browning marker uncoupling protein-1 (UCP1) was detected to assess the degree of lower limb adipocyte browning after 1 week, 2 weeks, and 4 weeks of mitochondrial transplantation (Figures 4A, 4B, and S5). In the first week, we could observe that there was no significant difference in the number of UCP1-positive cells between the mitochondrial transplantation group and the lower limb IRI group. In the second week, the expression of UCP1 in the mitochondrial transplantation group was significantly increased, which was 81.9% higher than that in the lower limb IRI group. In the fourth week, this value was 2.8 times higher than that in the model group. These results suggested that mitochondrial transplantation may play a role in promoting adipocyte browning in lower limb IRI.

It had been shown that the mitochondrial inner membrane protein optic atrophy-associated protein 1 (OPA1) can reduce lower limb IRI by promoting adipocyte browning.[27,30] Therefore, we speculate that mitochondrial transplantation may play a therapeutic role in lower limb IRI by promoting the increase of OPA1 and promoting adipocyte browning. To confirm this conjecture, OPA1 protein was detected in the tissues after 24 h and 48 h of intramuscular injection.

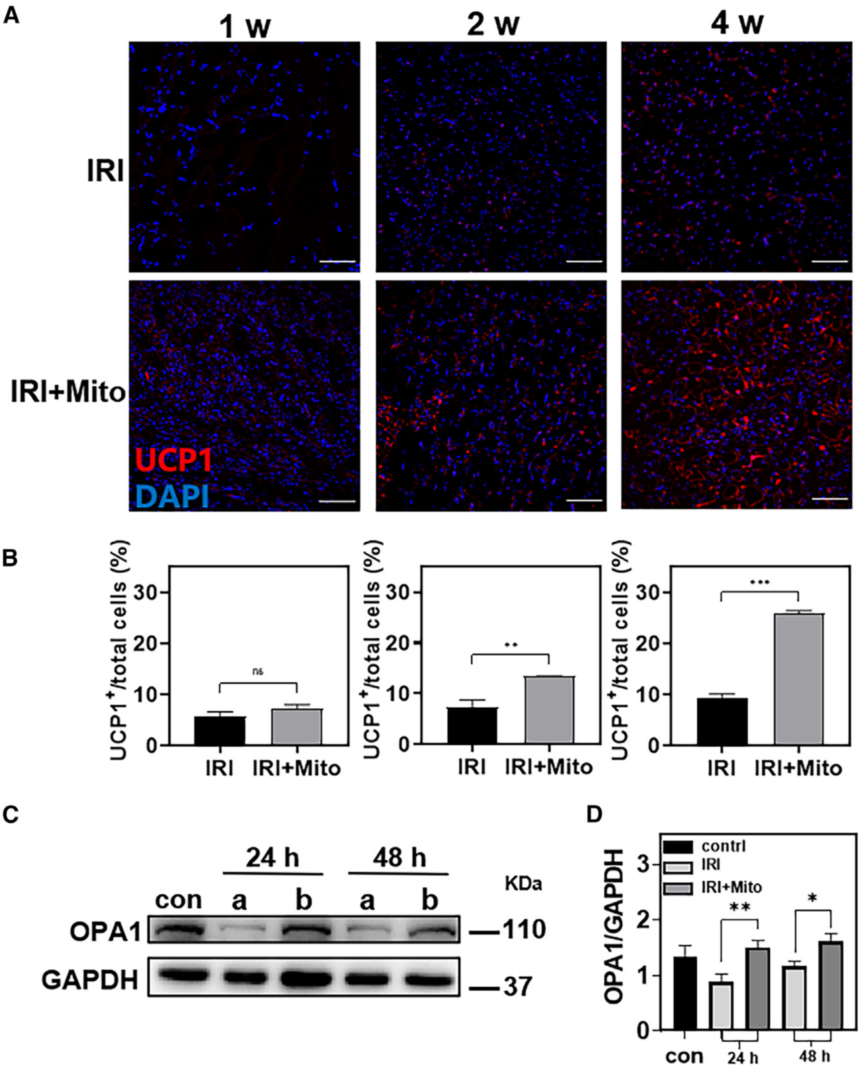

Figure 4. Mitochondrial transplantation promoting effects on the adipocyte browning

(A and B) Immunofluorescence staining CLSM images and related quantitative analysis were obtained after 1 week, 2 weeks, and 4 weeks in the lower limb (blue, nucleus; red, UCP1; scale bars, 100 μm). Statistical significance was analyzed using *t*-tests. (C and D) Western blotting and related quantitative analysis of OPA1 in the tissues after 24 h and 48 h. (a) Lower limb IRI, (b) lower limb IRI + Mito. Statistical significance was analyzed using the Kruskal-Wallis test. Experimental data are mean ± SD of samples in a representative experiment (n = 3). Asterisk (*) denotes statistical significance between bars (*$p < 0.05$, **$p < 0.01$, ***$p < 0.001$).

As shown in Figures 4C, 4D, and S12B, mitochondrial transplantation increased OPA1 by 0.7 times compared with the lower limb IRI group after 24 h of treatment. And at 48 h, the OPA1 in the mitochondrial transplantation group was 1.4 times higher than that in the lower limb IRI group. These results might indicate that mitochondrial transplantation increased OPA1.

Effects of mitochondrial transplantation on skeletal muscle cells *in vitro*

Based on the results of *in vivo* studies, we speculated that transplanted mitochondrial may be taken up by adipocytes while supplementing mitochondria for damaged skeletal muscle cells, and promoting tissue repaired by increasing OPA1 to promote adipocyte browning. To further verify this hypothesis, we first induced myoblast C2C12 to differentiate into skeletal muscle cells *in vitro* and studied their uptake behavior of mitochondria. As shown in Figures 5A, 5B, and S6, the original mitochondria of C2C12 cells were labeled with green fluorescence and the mitochondria of hMSCs were labeled with red fluorescence. After incubation for different times, it was observed that the red fluorescence in C2C12 cells gradually increased with the extension of time, indicating that the internalized mitochondria were gradually increasing. In addition, the colocalization of red and green fluorescence is gradually obvious, which is consistent with the phenomenon observed in other studies related to mitochondrial transplantation.[17,41] It suggested that the donor mitochondria may fuse with receptor mitochondria to supplement the function of original mitochondria. Therefore, we further examined the production of ATP in C2C12 cells after the incubation with the mitochondria of hMSCs. As shown in Figures 5C and 5D, the production of ATP in C2C12 cells was 1.4 times higher than that in the model group after 3 h of mitochondrial transplantation. This value was 2.5 times in the 24 h of mitochondrial transplantation group, which proved that transplanted mitochondria could supplement the function of mitochondrial in IRI skeletal muscle cells.

Effect of mitochondrial transplantation on adipocyte browning *in vitro*

We also studied the uptake of mitochondria by mature adipocytes induced from 3T3-L1 cells. Similar to C2C12, in Figures 6A, 6B, and S7, the internalized mitochondria increased with the extension of time, and the colocalization with the mitochondria of adipocytes gradually increased. Moreover, consistent with the results in *vivo*, mitochondrial transplantation significantly increased the protein expression of OPA1 in mature 3T3-L1 adipocytes (Figures 6C, 6D, and S12C). Furthermore, we examined the effect of mitochondrial transplantation on adipocyte browning. Taking mature 3T3-L1 adipocytes without mitochondrial transplantation as negative control and mirabegron (i.e., 3T3-L1 were treated with 3 μg/mL mirabegron for 6 h) induction as positive control,[42] the protein expression of

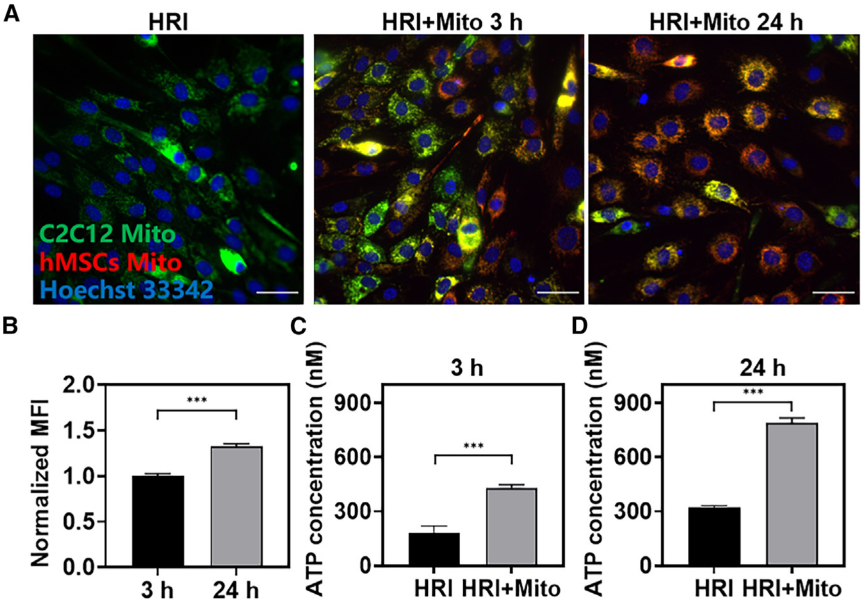

Figure 5. *In vitro* effects of mitochondrial transplantation on skeletal muscle cells
(A and B) CLSM images of mitochondrial uptake by C2C12 cells at 3 h and 24 h after hypoxia and reoxygenation (blue, nucleus; green, original mitochondria of C2C12 cells; red, mitochondria of hMSCs; scale bars, 50 μm). (C and D) ATP production at 3 h and 24 h in C2C12 cells in the hypoxia-reoxygenation model group and mitochondrial transplantation treatment group. Statistical significance was analyzed using *t*-tests. Experimental data are mean ± SD of samples in a representative experiment (n = 3). Asterisk (*) denotes statistical significance between bars (***p < 0.001). HRI, hypoxia-reoxygenation injury.

UCP1 was significantly increased after mitochondrial transplantation (Figures 6E, 6F and S12D). Immunofluorescence assay of UCP1 had similar results (Figures 6G, 6H, and S8). Meanwhile, mitochondrial membrane potential was detected by the JC-1 probe, and it was observed that the mitochondrial membrane potential in the mitochondrial transplantation group and the mirabegron-induced group were increased compared with the negative control group, the ratio of the intensity of red fluorescence to green fluorescence was increased, which could also demonstrate the enhanced mitochondrial activity in adipocytes. (Figures 6I, 6J, and S9). Since one of the typical characteristics of mature brown adipocytes is high mitochondrial content and activity, this result also proved that mitochondrial transplantation promoted adipocyte browning from another point of view. In contrast, Oil Red O staining (Figures 6K and 6L) also showed that mitochondrial transplantation reduced the number of white adipocytes. These results suggested that mitochondrial transplantation might increase the protein expression of OPA1 in adipocytes and promote adipocyte browning, which may alleviate the lower limb IRI.

Small interfering RNA interferes with the expression of OPA1 and UCP1 in 3T3-L1 and inhibits the process of adipocyte browning

To further investigate the role of OPA1 in adipocyte browning, we silenced OPA1 using small interfering RNA (siRNA) in 3T3-L1 cells. First, the interference efficiency of the four pairs of siRNAs against OPA1 was verified by western blotting. Good interference effect on OPA1 in 3T3-L1 were si-OPA1-2 and si-OPA1-4, which were used in subsequent experiments (Figure S10). We performed experiments on four different groups: the blank group, si-OPA1, mitochondrial transplantation, and the combined si-OPA1 and mitochondrial group (transfection with si-OPA1 followed by transplantation of mitochondria), in which a basal level of OPA1 expression, in the si-OPA1 group, OPA1 expression was inhibited by siRNA transfection. In the mitochondrial transplantation group, OPA1 expression was significantly elevated, indicating that mitochondrial transplantation increased the expression of OPA1 (Figures 7A, 7B, and S12E; Table S2). Next, the expression of UCP1 in each group was analyzed by western blotting and immunofluorescent staining, and in the blank group, UCP1 expression was very little, indicating the absence of spontaneous adipocyte browning. In the si-OPA1 group that inhibited the expression of OPA1, the expression of UCP1 was significantly decreased compared with the blank group, indicating that the inhibition of OPA1 expression hindered the browning process. UCP1 expression was significantly upregulated in the mitochondrial transplantation group, suggesting that mitochondrial transplantation can promote adipocyte browning and induce UCP1 expression. In the si-OPA1 and mitochondrial combination group, UCP1 expression was significantly decreased compared with the mitochondrial transplantation group, indicating that OPA1 silencing attenuated the promoting effect of mitochondrial transplantation on UCP1 expression (Figures 7C–7F, S11, and S12F). These results suggest that OPA1 may play a crucial role in regulating adipocyte browning, as mitochondrial transplantation alone promoted UCP1 expression and adipocyte browning, but this effect was attenuated when OPA1 expression was silenced.

To further investigate the role of UCP1 in adipocyte browning, we knocked down UCP1 *in vitro*. The results demonstrated that knockdown of UCP1 barely affected lipid loading of adipocytes. At the same time, mitochondrial transplantation could not decrease the lipid loading in the adipocytes with knockdown of UCP1. In contrast, mitochondrial transplantation reduced the amount of lipid droplets in the adipocytes without knockdown of UCP1(Figure S13 and Table S3). It can be seen that UCP1 is an important factor in the process of adipocyte browning caused by mitochondrial transplantation.

DISCUSSION

In recent years, there have been various therapeutic measures targeting mitochondria to improve ischemic diseases.[43,44] Enhancing cellular

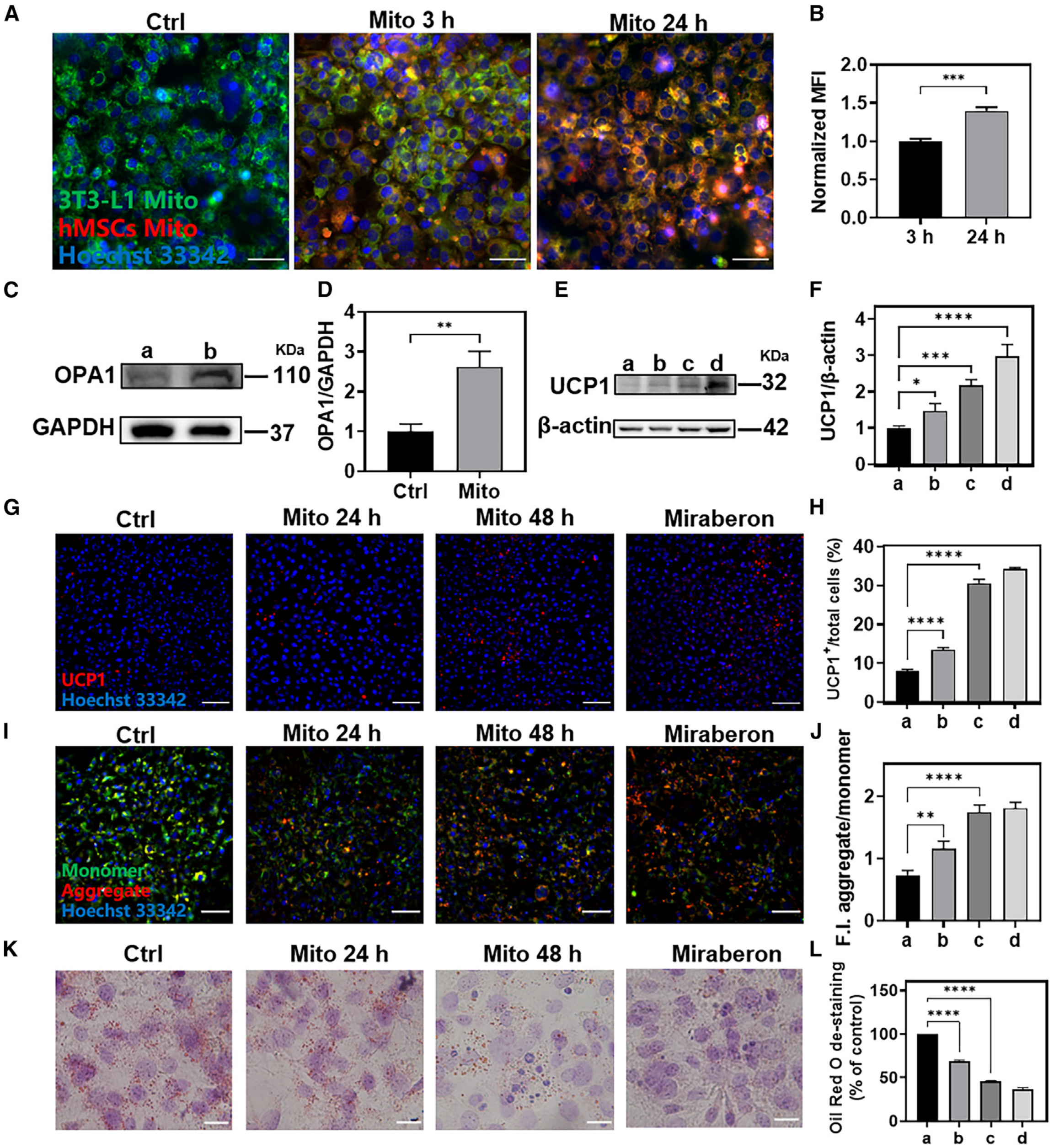

Figure 6. ***In vitro*** **effects of mitochondrial transplantation on adipocyte browning**

(A) CLSM images of mitochondrial uptake by 3T3-L1 cells at 3 h and 24 h (blue, nucleus; green, 3T3-L1 cell mitochondria; red, hMSCs-derived mitochondria; scale bars, 50 μm). (B) Quantitative analysis of red fluorescence in (A). (C and D) OPA1 protein expression after 24 h of mitochondrial transplantation. (a) Control (Ctrl) mature adipocytes. (b) Mito, mature adipocytes transplanted with mitochondrial. Statistical significance was analyzed using *t*-tests. (E and F) Western blotting analysis of UCP1 protein expression. (G and H) CLSM images of UCP1 immunofluorescence (blue, nucleus; red, UCP1; scale bars, 100 μm). (I and J) JC-1 staining CLSM image and quantitative analysis of red fluorescence/green fluorescence in (I) (blue:, nucleus; green, JC-1 monomer; red, JC-1 aggregate; scale bars, 100 μm). (K and L) Oil Red O staining and quantification (scale bar, 10 μm). Samples for (H), (J), and (L): (a) Ctrl, mature adipocytes, (b) mature adipocytes transplanted with mitochondria for 24 h, (c) mature adipocytes transplanted with mitochondria for 48 h, (d) mature adipocytes treated with Mirabegron. Statistical significance was analyzed using one-way ANOVA. Experimental data are mean ± SD of samples in a representative experiment (n = 3). Asterisk (*) denotes statistical significance between bars (*p < 0.05, **p < 0.01, ***p < 0.001, ****p < 0.0001).

mitochondrial efficacy include the following three main pathways: protecting/enhancing the efficacy of pre-existing mitochondria by exotic drugs[45,46]; promoting mitochondrial biogenesis through foreign drugs[47]; and transplantation of exogenous mitochondria. Because mitochondrial transplantation improves the level of cellular metabolism in general, rather than partially targeting a single pathway, it has become

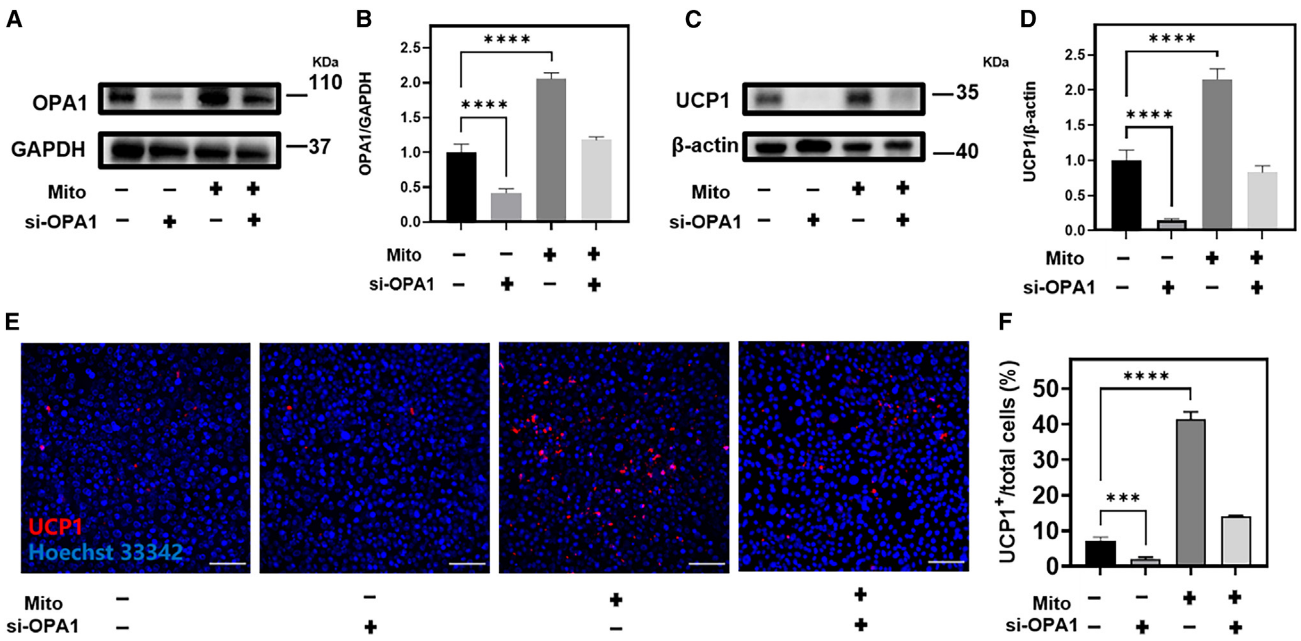

Figure 7. siRNA interferes with the expression of OPA1 in 3T3-L1 and inhibits the process of adipocyte browning

(A–D) The protein levels of OPA1 and UCP-1 in 3T3-L1 cell blank group, si-OPA1 group, mitochondrial transplantation group, OPA1 silenced first and then added mitochondria group after 48 h of treatment respectively. (E and F) UCP1 immunofluorescence staining and quantification of 3T3-L1 cell blank group, si-OPA1 group, mitochondrial transplantation group, first silencing OPA1 and then adding mitochondria group after 48 h of treatment (blue, nucleus; red, UCP1; scale bars, 100 μm). Statistical significance was analyzed using one-way ANOVA. Experimental data are mean ± SD of samples in a representative experiment (n = 3). Asterisk (*) denotes statistical significance between bars (***p < 0.001, ****p < 0.0001).

an important direction in the treatment of ischemic diseases.[48] It has been reported that mitochondrial transplantation can reduce the extent of muscle infarction and improve muscle function, effectively reducing the incidence of lower limb IRI,[37] and our study provides a perspective for understanding the possible mechanisms by which mitochondrial transplantation protects against lower limb ischemia.

By conducting transplantation of hMSCs-derived mitochondria into the damaged gastrocnemius muscle in mice, we observed remarkable outcomes. The transplanted mitochondria were taken up by both skeletal muscle cells and adipocytes, leading to an increase in the expression of OPA1 and UCP1. These findings provide valuable insights into a potential therapeutic mechanism of mitochondrial transplantation in the treatment of lower limb IRI.

However, despite the promising results, several aspects warrant further investigation. First, it would be valuable to generate OPA1 knockout mice and UCP1 knockout mice to confirm the specific role of OPA1 and UCP1 in the observed adipocyte browning effect following mitochondrial transplantation. Additionally, while our study focused on the uptake of mitochondria by skeletal muscle cells and adipocytes, exploring the uptake by other cell subpopulations would provide a more comprehensive understanding of the cellular interactions involved. Moreover, addressing the technical challenges associated with the clinical application of isolated mitochondria is crucial for future translation.

In conclusion, our study provides compelling evidence supporting the therapeutic potential of hMSCs-derived mitochondrial transplantation in repairing lower limb IRI. Beneficial effects of mitochondrial transplantation were observed reduction in cell apoptosis and promotion of ATP production in skeletal muscle cells. Furthermore, the transformation of the adipocyte phenotype suggests that an additional mechanism by which mitochondrial transplantation may alleviate lower limb IRI, suggesting that other subpopulation of receptor cells is just as important as the classical primary subpopulation of recipient cells alleviating lower limb IRI. Future studies addressing the remaining questions and optimizing the use of mitochondrial transplantation as a clinical intervention hold great promise.

MATERIALS AND METHODS

Mitochondrial isolated of hMSCs

According to the instructions of the mitochondrial isolated kit (Thermo, #89874),[49] the cells were collected and centrifuged at 850×*g* for 2 min. The cells were precipitated and resuspended with separation reagent A solution and incubated on ice for 2 min 10 μL separation reagent B was added and incubated for 5 min on ice, vortexing once per minute. We then added 800 μL separation reagent C, mix upside down, and centrifuge at 700×*g* at 4°C for 10 min. The supernatant was transferred to a new 2-mL centrifuge tube and centrifuged at 12,000×*g* at 4°C for 15 min. Collect the supernatant (at this point, the supernatant is cytosolic); 500 μL separation reagent C was

added to the precipitate, resuspended, and centrifuged at 12,000×g for 5 min at 4°C. The supernatant was discarded and resuspended in PBS to obtain complete mitochondria derived from mesenchymal stem cells.

Characterization of hMSCs-derived mitochondria

Mitochondria in and out of cells were labeled with Mito Tracker Deep Red FM (Cell Signaling Technology #8778) at concentrations of 250 nM and 100 nM, respectively, and the fluorescence images before and after mitochondrial isolation were observed under an inverted fluorescence microscope (Leica Microsystems, SN529001). Nanosight detects the size and concentration of mitochondria isolated from 2×10^7 hMSCs.[50] TEM (JEM-2100, Hitachi) was used to analyze the morphology and size of mitochondria before and after isolation.[51] The mitochondrial and cytoplasmic proteins β-actin (Abcam, ab8226), Cytrate synthase (abcepta, AP5894a), TOM20 (abcepta, AP59783), COX4(abcepta, AP22111a), and cytochrome c (abcepta, AP20772c) were detected by Western blot, and the integrity and purity of the isolated mitochondria were evaluated. JC-1 probe (FMS-FZ006, FcMACS) was incubated with Mito and Mito + Antimycin A (i.e., mitochondria were treated with 1 mM antimycin A for 30 min) at room temperature for 15 min. After PBS washing and centrifugation, the absorbance was detected by microplate analyzer (N12639-02, Thermo).[35] The normal mitochondria at the concentration of 5.8×10^8/mL were mixed with the substrate glutamate (0.4 mM) and malate (0.2 mM), then ADP (0.01 mM) was added, and the operation was performed according to the instruction of ATP detection kit (Thermo, A22066).[36] Chemiluminescence intensity was measured with a multifunctional microplate reader (PerkinElmer EnVision).

Animal experiments

All animal experiments were performed in strict accordance with the guidelines of the National Institutes of Health Animal Care and Use Committee and were approved by the Animal Experimentation Ethics Committee of Nanjing Drum Tower Hospital, ethics number: DWSY-22068194. Male C57/BL6 mice (10–12 weeks) were provided by Biotechnology Co., Ltd. The mice were housed in a stable room temperature environment with an ambient temperature of 22°C throughout the study. Additionally, their food intake was fixed and carefully monitored to ensure no significant differences in caloric intake between the experimental groups. The mice were provided with a standard diet, and their food intake was recorded daily. To simulate acute limb IRI, mice were anesthetized by inhalation of 2%–3% isoflurane and maintained. After 3 h of ischemia, the right hindlimb of C57/BL6 mice was ligated with a 3M rubber band (3.5 oz), the tourniquet was removed and the mice were restored for 15 min,[52] then, mice in the treatment group were injected with 50 μL Mito Tracker Deep Red FM-labeled live hMSCs-derived mitochondria at a concentration of 2×10^8/mL in the gastrocnemius muscle.

Therapeutic effects *in vivo*

Male C57/BL6 mice (10–12 weeks) were randomly divided into three groups: control group (completely healthy or contralateral healthy muscle of model group), model group, and mitochondrial transplantation treatment group. For TUNEL apoptosis, the sample size was 18 (healthy, model and treatment time points were 24 h and 48 h, respectively). For histomorphological evaluation, hematoxylin and eosin staining, Masson staining, and Wolf scarlet staining were performed with a sample size of 18 (healthy, model and treatment time points were 2 weeks and 4 weeks, respectively). For evaluating mouse gait, the sample size was eight (for modeling, the treatment time points were 24 h, 1 week, 2 weeks, and 4 weeks, respectively).

Immunofluorescence of mitochondrial uptake and adipocyte browning in mice

Skeletal muscle cells were labeled with anti-myogenin (Abcam, AB1835),[53] adipocytes with anti-adiponectin (Abcam, AB181281),[54] and formation of brown adipocytes with anti-UCP1 (Servicebio, GB112174). The nuclei were labeled with DAPI (Servicebio, G1012), and imaged by confocal laser scanning fluorescence microscopy (CLSM), and the mean fluorescence intensity was analyzed by ImageJ software.

Cell culture and differentiation

hMSCs (ATCC, CP-CL11) were cultured in a complete medium containing 90% DMEM/F12, 10% fetal bovine serum (FBS), and penicillin/streptomycin. The culture was incubated at 37°C in a humid environment containing 5% CO_2. Mouse myoblasts C2C12 (Human Fenghui Biotechnology Co., Ltd) were cultured in a complete medium containing 90% DMEM (Sperikon Life Science & Biotechnology Co., Ltd), 10% FBS, and penicillin/streptomycin before differentiation. The cells were cultured at 37°C in a humid environment containing 5% CO_2, and when the cells grew to 80%, they were replaced with a differentiation medium (2%HBS, 1% penicillin/streptomycin DMEM medium).[55] Cultured to mature skeletal muscle cells with myotubes, mouse embryonic fibroblasts 3T3-L1(Human Fenghui Biotechnology Co., Ltd) were cultured in a complete medium containing 90% DMEM, 10% calf serum (NBCS) and penicillin/streptomycin, in a moist environment with 5% CO_2 at 37° C. When the cells grew to 80%, NBCS was replaced with FBS and treated with 10 μg/mL bovine insulin (source leaf, S12033), 0.5 mM 3-isobutyl-1-methylxanthine (Source Leaf, B28582) and 1 μM dexamethasone (Solarbio, D8040) for 2 days. Then they were incubated in a medium containing 10 μg/mL insulin for another 2 days. Thereafter, the differentiation medium was replaced with basal growth medium every other day until it was induced into mature adipocytes.[56]

Cell modeling and therapy

After differentiation, C2C12 cells were changed into a sugar-free medium and placed in an anaerobic culturing bag (Anaeropack) (0.1% oxygen). After culturing at 37°C for 1 h, C2C12 cells were changed back to a complete medium. In the treatment group, 1×10^8 mitochondria were added to each well.[57]

In vitro mitochondrial uptake, adipocyte browning, and staining of JC-1

For mitochondrial uptake, after the completion of cell differentiation, the cells were washed twice with PBS, stained with mito-tracker Green

(Beyotime, C1048) for 30 min in the dark, washed twice with PBS, and then simulated IRI with anaerobic culture bag (0.1% oxygen) for modeling. After 1 h of modeling, adding Mito Tracker Deep Red FM prestained mitochondria for 3 h and 24 h, respectively. Hoechst 33342 live cell staining solution (Biosharp, BL803A) was used to label the nuclei, and then the mitochondrial uptake was photographed by CLSM. The mean fluorescence intensity was analyzed by ImageJ software. For adipocyte browning, cultured cells were fixed with 4% paraformaldehyde for 20 min, permeated with 0.2% Triton X-100 for 10 min, and then blocked with 5% BSA for 30 min after adding pre-stained mitochondria for 24 h and 48 h. The cells were incubated with anti-UCP1 (Cell Signaling, 722985) in a wet box at 4°C overnight, and the secondary antibodies were incubated with cells at room temperature for 2 h (protected from light). The nuclei were stained with Hoechst33342 for 10 min and photographed by CLSM. For JC-1, 1× JC-1 working solution, the samples were incubated at 37°C for 15–60 min, and the CLSM photos were taken after Hoechst33342 standing for 10 min.

In vitro ATP production

At 3 h and 24 h after C2C12 cell modeling treatment, cell lysates were collected and operated according to the instruction of the ATP detection kit, and the chemiluminescence intensity was detected by a multi-function microplate analyzer.[36]

In vitro oil red O staining

After adding mitochondria for 24 h and 48 h, the cells were operated according to Biyuntian modified Oil Red O staining kit (C0158S). The cells were fixed with 4% paraformaldehyde for 10 min and covered with staining detergent for 20 s. Oil red O staining working solution for 10–20 min; the washing solution was allowed to stand for 30 s. The staining washing solution was removed and washed with PBS. Hematoxylin was counterstained for 1 min; then we performed distilled water washing. We observed and took pictures under a microscope. The proportion of white adipocytes was analyzed by ImageJ software.[58]

Western blotting analysis of OPA1 in adipocyte browning

For mouse tissues, 24 h and 48 h after mouse modeling treatment, the hindlimb muscles were taken, RIPA lysate was added, and ground with a frozen tissue lapping instrument (JXFSTPRP-CL, Shanghai), centrifuged at 12,000×*g* for 15 min, and the supernatant was taken, and BCA protein quantification kit (ElabScience, E-bc-k318-m) to detect the protein concentration; For 3T3-L1 cells, proteins were isolated 24 h and 48 h after the addition of mitochondria, electrophoresed, and transferred to nitrocellulose membranes according to the standard protocol. A washing buffer and antibody solution were prepared with TBST. After 2 h of the blockade in 5% BSA (Aladdin, A116563), the membranes were mixed with anti-OPA1 (Abcepta, AP20727c), UCP1 (Cell Signaling, 722985), β-actin (Abcam, AB8226), and GAPDH (Abcam, AB8245) were incubated overnight at 4°C, followed by incubation with appropriate secondary antibodies for 2 h at room temperature. Enhanced chemiluminescence (Tanon 5200 Multi, China) was used to detect the signal, and ImageJ software was used to analyze the protein bands.

siRNA-mediated silencing of OPA1 in 3T3-L1 cells

3T3-L1 were differentiated into mature adipocytes, and cells were transfected with siRNA targeting OPA1 (Shanghai GenePharma Co., Ltd).[59,60] Dilute siRNA and Transfect-Mate (Shanghai GenePharma Co., Ltd) in DMEM basal medium according to the final concentrations recommended in the instructions. The mixture was allowed to stand at room temperature for 5 min. Subsequently, si-OPA1 and Transfect-Mate were combined and allowed to stand for an additional 20 min at room temperature. Add the mixture dropwise to the 3T3-L1 culture system to be transfected. Eight hours after transfection, the complete medium was changed to allow continued cell culture. After 48–72 h of transfection, the cells were collected to verify the interference efficiency by western blotting. Each group was divided into 3T3-L1 cell blank group, si-OPA1 group, and mitochondrial transplantation group. OPA1 was first silenced and then mitochondria were transplanted for 48 h. Then the expression of OPA1 and UCP1 in each group was detected by western blot and immunofluorescence.

siRNA-mediated silencing of UCP1 in 3T3-L1 cells

3T3-L1 were differentiated into mature adipocytes, and cells were transfected with siRNA targeting UCP1 (Shanghai GenePharma Co., Ltd). Dilute siRNA and Transfect-Mate (Shanghai GenePharma Co., Ltd) in DMEM basal medium according to the final concentrations recommended in the instructions. The mixture was allowed to stand at room temperature for 5 min. Subsequently, si-UCP1 and Transfect-Mate were combined and allowed to stand for an additional 20 min at room temperature. Add the mixture dropwise to the 3T3-L1 culture system to be transfected. Eight hours after transfection, the complete medium was changed to allow continued cell culture. After 48–72 h of transfection, the cells were collected to verify the interference efficiency by western blotting. Each group was divided into 3T3-L1 cell blank group, si-UCP1 group, and mitochondrial transplantation group. UCP1 was first silenced and then mitochondria were transplanted for 48 h. Then the expression of UCP1 in each group was detected by western blot and oil red O staining.

Statistical analysis

Analyses were performed using SPSS 18.0 software: comparisons of the two groups were performed by *t*-test (normally distributed data), Wilcoxon test (non-normally distributed data); comparisons of three or more groups were performed by one-way ANOVA (normally distributed data), Kruskal-Wallis test (non-normally distributed data). A p value of ≤ 0.05 was considered significant. Statistics were calculated using Prism (GraphPad Software). All data are shown as mean ± SD.

DATA AND CODE AVAILABILITY

Internet access to original recordings and images will be provided upon request.

SUPPLEMENTAL INFORMATION

Supplemental information can be found online at https://doi.org/10.1016/j.omtm.2023.101152.

ACKNOWLEDGMENTS

This work is funded by the National Nature Science Foundation of China (No. 82272098).

AUTHOR CONTRIBUTIONS

Experiments were designed by J.Z., L.Z., H.N., Z.W., and M.Z.; methodology was perfected by J.Z., J.L., H.N., L.Z., J.W., Y.L., W.J., Z.W., and M.Z.; experimental work and visualization were conducted by J.Z., J.L., H.N., L.Z., J.W., and Y.L.; funding was acquired by M.Z.; the project was administered by Z.W. and M.Z.; supervision was conducted by Z.W. and M.Z.; J.Z. wrote the original draft; manuscript review and editing were completed by J.Z., J.L., H.N., L.Z., J.W., Y.L., W.J., Z.W., and M.Z.

DECLARATION OF INTERESTS

The authors declare no competing or other financial interests.

REFERENCES

1. Kurian, G.A., Rajagopal, R., Vedantham, S., and Rajesh, M. (2016). The Role of Oxidative Stress in Myocardial Ischemia and Reperfusion Injury and Remodeling: Revisited. Oxid. Med. Cell. Longev. *2016*, 1656450.
2. Duehrkop, C., Banz, Y., Spirig, R., Miescher, S., Nolte, M.W., Spycher, M., Smith, R.A.G., Sacks, S.H., and Rieben, R. (2013). C1 esterase inhibitor reduces lower extremity ischemia/reperfusion injury and associated lung damage. PLoS One *8*, e72059.
3. Beckman, J.A., Schneider, P.A., and Conte, M.S. (2021). Advances in Revascularization for Peripheral Artery Disease: Revascularization in PAD. Circ. Res. *128*, 1885–1912.
4. Shadel, G.S., and Horvath, T.L. (2015). Mitochondrial ROS signaling in organismal homeostasis. Cell *163*, 560–569.
5. Pipinos, I.I., Swanson, S.A., Zhu, Z., Nella, A.A., Weiss, D.J., Gutti, T.L., McComb, R.D., Baxter, B.T., Lynch, T.G., and Casale, G.P. (2008). Chronically ischemic mouse skeletal muscle exhibits myopathy in association with mitochondrial dysfunction and oxidative damage. Am. J. Physiol. Regul. Integr. Comp. Physiol. *295*, R290–R296.
6. Lynn, E.G., Lu, Z., Minerbi, D., and Sack, M.N. (2007). The regulation, control, and consequences of mitochondrial oxygen utilization and disposition in the heart and skeletal muscle during hypoxia. Antioxid. Redox Signal. *9*, 1353–1361.
7. Sun, J., Lo, H.T.J., Fan, L., Yiu, T.L., Shakoor, A., Li, G., Lee, W.Y.W., and Sun, D. (2022). High-efficiency quantitative control of mitochondrial transfer based on droplet microfluidics and its application on muscle regeneration. Sci. Adv. *8*, eabp9245.
8. Romanello, V., and Sandri, M. (2015). Mitochondrial Quality Control and Muscle Mass Maintenance. Front. Physiol. *6*, 422.
9. Ryan, T.E., Schmidt, C.A., Green, T.D., Brown, D.A., Neufer, P.D., and McClung, J.M. (2015). Mitochondrial Regulation of the Muscle Microenvironment in Critical Limb Ischemia. Front. Physiol. *6*, 336.
10. Guillot, M., Charles, A.L., Lejay, A., Pottecher, J., Meyer, A., Georg, I., Goupilleau, F., Diemunsch, P., and Geny, B. (2021). Deleterious Effects of Remote Ischaemic Perconditioning During Lower Limb Ischaemia-Reperfusion in Mice. Eur. J. Vasc. Endovasc. Surg. *62*, 953–959.
11. Kawamura, I., Takemura, G., Kanamori, H., Takeyama, T., Kawaguchi, T., Tsujimoto, A., Goto, K., Maruyama, R., Watanabe, T., Shiraki, T., et al. (2010). Repeated phlebotomy augments angiogenesis to improve blood flow in murine ischemic legs. Am. J. Physiol. Heart Circ. Physiol. *299*, H372–H378.
12. Rosero, O., Németh, K., Turóczi, Z., Fülöp, A., Garbaisz, D., Győrffy, A., Szuák, A., Dorogi, B., Kiss, M., Nemeskéri, Á., et al. (2014). Collateral circulation of the rat lower limb and its significance in ischemia-reperfusion studies. Surg. Today *44*, 2345–2353.
13. Olguner, C., Koca, U., Kar, A., Karci, A., Işlekel, H., Canyilmaz, M., Mavioĝlu, O., Kizildağ, S., Unlü, G., and Elar, Z. (2006). Ischemic preconditioning attenuates the lipid peroxidation and remote lung injury in the rat model of unilateral lower limb ischemia reperfusion. Acta Anaesthesiol. Scand. *50*, 150–155.
14. Apichartpiyakul, P., Shinlapawittayatorn, K., Rerkasem, K., Chattipakorn, S.C., and Chattipakorn, N. (2022). Mechanisms and Interventions on Acute Lower Limb Ischemia/Reperfusion Injury: A Review and Insights from Cell to Clinical Investigations. Ann. Vasc. Surg. *86*, 452–481.
15. Sun, X., Gao, R., Li, W., Zhao, Y., Yang, H., Chen, H., Jiang, H., Dong, Z., Hu, J., Liu, J., et al. (2021). Alda-1 treatment promotes the therapeutic effect of mitochondrial transplantation for myocardial ischemia-reperfusion injury. Bioact. Mater. *6*, 2058–2069.
16. Moskowitzova, K., Orfany, A., Liu, K., Ramirez-Barbieri, G., Thedsanamoorthy, J.K., Yao, R., Guariento, A., Doulamis, I.P., Blitzer, D., Shin, B., et al. (2020). Mitochondrial transplantation enhances murine lung viability and recovery after ischemia-reperfusion injury. Am. J. Physiol. Lung Cell Mol. Physiol. *318*, L78–L88.
17. Xie, Q., Zeng, J., Zheng, Y., Li, T., Ren, J., Chen, K., Zhang, Q., Xie, R., Xu, F., and Zhu, J. (2021). Mitochondrial Transplantation Attenuates Cerebral Ischemia-Reperfusion Injury: Possible Involvement of Mitochondrial Component Separation. Oxid. Med. Cell. Longev. *2021*, 1006636.
18. Kubat, G.B., Kartal, Y., Atalay, O., Ulger, O., Ekinci, O., Celik, E., Safali, M., Urkan, M., Karahan, S., Ozler, M., et al. (2021). Investigation of the effect of isolated mitochondria transplantation on renal ischemia-reperfusion injury in rats. Toxicol. Appl. Pharmacol. *433*, 115780.
19. Park, A., Oh, M., Lee, S.J., Oh, K.J., Lee, E.W., Lee, S.C., Bae, K.H., Han, B.S., and Kim, W.K. (2021). Mitochondrial Transplantation as a Novel Therapeutic Strategy for Mitochondrial Diseases. Int. J. Mol. Sci. *22*, 4793.
20. Valenti, D., Vacca, R.A., Moro, L., and Atlante, A. (2021). Mitochondria Can Cross Cell Boundaries: An Overview of the Biological Relevance, Pathophysiological Implications and Therapeutic Perspectives of Intercellular Mitochondrial Transfer. Int. J. Mol. Sci. *22*, 8312.
21. Ikeda, G., Santoso, M.R., Tada, Y., Li, A.M., Vaskova, E., Jung, J.H., O'Brien, C., Egan, E., Ye, J., and Yang, P.C. (2021). Mitochondria-Rich Extracellular Vesicles From Autologous Stem Cell-Derived Cardiomyocytes Restore Energetics of Ischemic Myocardium. J. Am. Coll. Cardiol. *77*, 1073–1088.
22. Lin, H.C., Liu, S.Y., Lai, H.S., and Lai, I.R. (2013). Isolated mitochondria infusion mitigates ischemia-reperfusion injury of the liver in rats. Shock *39*, 304–310.
23. Huang, T., Zhang, T., Jiang, X., Li, A., Su, Y., Bian, Q., Wu, H., Lin, R., Li, N., Cao, H., et al. (2021). Iron oxide nanoparticles augment the intercellular mitochondrial transfer-mediated therapy. Sci. Adv. *7*, eabj0534.
24. Tseng, N., Lambie, S.C., Huynh, C.Q., Sanford, B., Patel, M., Herson, P.S., and Ormond, D.R. (2021). Mitochondrial transfer from mesenchymal stem cells improves neuronal metabolism after oxidant injury in vitro: The role of Miro1. J. Cereb. Blood Flow Metab. *41*, 761–770.
25. Chen, W., Huang, J., Hu, Y., Khoshnam, S.E., and Sarkaki, A. (2020). Mitochondrial Transfer as a Therapeutic Strategy Against Ischemic Stroke. Transl. Stroke Res. *11*, 1214–1228.
26. Wang, J., Li, H., Yao, Y., Zhao, T., Chen, Y.Y., Shen, Y.L., Wang, L.L., and Zhu, Y. (2018). Stem cell-derived mitochondria transplantation: a novel strategy and the challenges for the treatment of tissue injury. Stem Cell Res. Ther. *9*, 106.
27. Bean, C., Audano, M., Varanita, T., Favaretto, F., Medaglia, M., Gerdol, M., Pernas, L., Stasi, F., Giacomello, M., Herkenne, S., et al. (2021). The mitochondrial protein Opa1 promotes adipocyte browning that is dependent on urea cycle metabolites. Nat. Metab. *3*, 1633–1647.
28. Pereira, R.O., Olvera, A.C., Marti, A., Fang, S., White, J.R., Westphal, M., Hewezi, R., AshShareef, S.T., García-Peña, L.M., Koneru, J., et al. (2022). OPA1 Regulates Lipid Metabolism and Cold-Induced Browning of White Adipose Tissue in Mice. Diabetes *71*, 2572–2583.
29. Pereira, R.O., Marti, A., Olvera, A.C., Tadinada, S.M., Bjorkman, S.H., Weatherford, E.T., Morgan, D.A., Westphal, M., Patel, P.H., Kirby, A.K., et al. (2021). OPA1 deletion in brown adipose tissue improves thermoregulation and systemic metabolism via FGF21. Elife *10*, e66519.

30. Zhang, H., Liu, M., Kim, H.T., Feeley, B.T., and Liu, X. (2021). Preconditioning improves muscle regeneration after ischemia-reperfusion injury. J. Orthop. Res. *39*, 1889–1897.

31. Yin, H., Pasut, A., Soleimani, V.D., Bentzinger, C.F., Antoun, G., Thorn, S., Seale, P., Fernando, P., van Ijcken, W., Grosveld, F., et al. (2013). MicroRNA-133 controls brown adipose determination in skeletal muscle satellite cells by targeting Prdm16. Cell Metabol. *17*, 210–224.

32. Lee, C., Liu, M., Agha, O., Kim, H.T., Liu, X., and Feeley, B.T. (2020). Beige fibro-adipogenic progenitor transplantation reduces muscle degeneration and improves function in a mouse model of delayed repair of rotator cuff tears. J. Shoulder Elbow Surg. *29*, 719–727.

33. Chen, W., Shi, K., Chu, B., Wei, X., and Qian, Z. (2019). Mitochondrial Surface Engineering for Multidrug Resistance Reversal. Nano Lett. *19*, 2905–2913.

34. Younis, A.Z., Lavery, G.G., Christian, M., and Doig, C.L. (2023). Rapid isolation of respiring skeletal muscle mitochondria using nitrogen cavitation. Front. Physiol. *14*, 1114595.

35. Suphim, B., Prawan, A., Kukongviriyapan, U., Kongpetch, S., Buranrat, B., and Kukongviriyapan, V. (2010). Redox modulation and human bile duct cancer inhibition by curcumin. Food Chem. Toxicol. *48*, 2265–2272.

36. Mitra, K., Wunder, C., Roysam, B., Lin, G., and Lippincott-Schwartz, J. (2009). A hyperfused mitochondrial state achieved at G1-S regulates cyclin E buildup and entry into S phase. Proc. Natl. Acad. Sci. USA *106*, 11960–11965.

37. Orfany, A., Arriola, C.G., Doulamis, I.P., Guariento, A., Ramirez-Barbieri, G., Moskowitzova, K., Shin, B., Blitzer, D., Rogers, C., Del Nido, P.J., and McCully, J.D. (2020). Mitochondrial transplantation ameliorates acute limb ischemia. J. Vasc. Surg. *71*, 1014–1026.

38. Yu, Z., Witman, N., Wang, W., Li, D., Yan, B., Deng, M., Wang, X., Wang, H., Zhou, G., Liu, W., et al. (2019). Cell-mediated delivery of VEGF modified mRNA enhances blood vessel regeneration and ameliorates murine critical limb ischemia. J. Contr. Release *310*, 103–114.

39. Owlarn, S., Klenner, F., Schmidt, D., Rabert, F., Tomasso, A., Reuter, H., Mulaw, M.A., Moritz, S., Gentile, L., Weidinger, G., and Bartscherer, K. (2017). Generic wound signals initiate regeneration in missing-tissue contexts. Nat. Commun. *8*, 2282.

40. Acín-Perez, R., Petcherski, A., Veliova, M., Benador, I.Y., Assali, E.A., Colleluori, G., Cinti, S., Brownstein, A.J., Baghdasarian, S., Livhits, M.J., et al. (2021). Recruitment and remodeling of peridroplet mitochondria in human adipose tissue. Redox Biol. *46*, 102087.

41. Sun, C., Liu, X., Wang, B., Wang, Z., Liu, Y., Di, C., Si, J., Li, H., Wu, Q., Xu, D., et al. (2019). Endocytosis-mediated mitochondrial transplantation: Transferring normal human astrocytic mitochondria into glioma cells rescues aerobic respiration and enhances radiosensitivity. Theranostics *9*, 3595–3607.

42. Hao, L., Scott, S., Abbasi, M., Zu, Y., Khan, M.S.H., Yang, Y., Wu, D., Zhao, L., and Wang, S. (2019). Beneficial Metabolic Effects of Mirabegron In Vitro and in High-Fat Diet-Induced Obese Mice. J. Pharmacol. Exp. Therapeut. *369*, 419–427.

43. Whitson, J.A., Bitto, A., Zhang, H., Sweetwyne, M.T., Coig, R., Bhayana, S., Shankland, E.G., Wang, L., Bammler, T.K., Mills, K.F., et al. (2020). SS-31 and NMN: Two paths to improve metabolism and function in aged hearts. Aging Cell *19*, e13213.

44. Bhargava, P., and Schnellmann, R.G. (2017). Mitochondrial energetics in the kidney. Nat. Rev. Nephrol. *13*, 629–646.

45. Yan, P., Chen, S.Q., Li, Z.P., Zhang, J., Xue, J.K., Wang, W.T., Huang, W.J., Cheng, J.Y., and Li, H.P. (2011). Effect of exogenous phosphocreatine on cardiomycytic apoptosis and expression of Bcl-2 and Bax after cardiopulmonary resuscitation in rats. World J. Emerg. Med. *2*, 291–295.

46. Dai, D.F., Hsieh, E.J., Chen, T., Menendez, L.G., Basisty, N.B., Tsai, L., Beyer, R.P., Crispin, D.A., Shulman, N.J., Szeto, H.H., et al. (2013). Global proteomics and pathway analysis of pressure-overload-induced heart failure and its attenuation by mitochondrial-targeted peptides. Circ. Heart Fail. *6*, 1067–1076.

47. Levay, M., Krobert, K.A., Wittig, K., Voigt, N., Bermudez, M., Wolber, G., Dobrev, D., Levy, F.O., and Wieland, T. (2013). NSC23766, a widely used inhibitor of Rac1 activation, additionally acts as a competitive antagonist at muscarinic acetylcholine receptors. J. Pharmacol. Exp. Therapeut. *347*, 69–79.

48. Masuzawa, A., Black, K.M., Pacak, C.A., Ericsson, M., Barnett, R.J., Drumm, C., Seth, P., Bloch, D.B., Levitsky, S., Cowan, D.B., and McCully, J.D. (2013). Transplantation of autologously derived mitochondria protects the heart from ischemia-reperfusion injury. Am. J. Physiol. Heart Circ. Physiol. *304*, H966–H982.

49. Zhang, Q., Raoof, M., Chen, Y., Sumi, Y., Sursal, T., Junger, W., Brohi, K., Itagaki, K., and Hauser, C.J. (2010). Circulating mitochondrial DAMPs cause inflammatory responses to injury. Nature *464*, 104–107.

50. Vultaggio-Poma, V., Falzoni, S., Chiozzi, P., Sarti, A.C., Adinolfi, E., Giuliani, A.L., Sánchez-Melgar, A., Boldrini, P., Zanoni, M., Tesei, A., et al. (2022). Extracellular ATP is increased by release of ATP-loaded microparticles triggered by nutrient deprivation. Theranostics *12*, 859–874.

51. Liu, J., Liu, X., Wu, M., Qi, G., and Liu, B. (2020). Engineering Living Mitochondria with AIE Photosensitizer for Synergistic Cancer Cell Ablation. Nano Lett. *20*, 7438–7445.

52. Crawford, R.S., Hashmi, F.F., Jones, J.E., Albadawi, H., McCormack, M., Eberlin, K., Entabi, F., Atkins, M.D., Conrad, M.F., Austen, W.G., Jr., and Watkins, M.T. (2007). A novel model of acute murine hindlimb ischemia. Am. J. Physiol. Heart Circ. Physiol. *292*, H830–H837.

53. Kim, M., Sung, B., Kang, Y.J., Kim, D.H., Lee, Y., Hwang, S.Y., Yoon, J.H., Yoo, M.A., Kim, C.M., Chung, H.Y., and Kim, N.D. (2015). The combination of ursolic acid and leucine potentiates the differentiation of C2C12 murine myoblasts through the mTOR signaling pathway. Int. J. Mol. Med. *35*, 755–762.

54. Shang, G.K., Han, L., Wang, Z.H., Song, M., Wang, D., Tan, Y.M., Li, Y.H., Li, Y.L., Zhang, W., and Zhong, M. (2021). Pim1 knockout alleviates sarcopenia in aging mice via reducing adipogenic differentiation of PDGFRalpha(+) mesenchymal progenitors. J. Cachexia Sarcopenia Muscle *12*, 1741–1756.

55. Wang, Y., Liu, S., Yan, Y., Li, S., and Tong, H. (2019). SPARCL1 promotes C2C12 cell differentiation via BMP7-mediated BMP/TGF-beta cell signaling pathway. Cell Death Dis. *10*, 852.

56. Yokomori, N., Tawata, M., and Onaya, T. (2002). DNA demethylation modulates mouse leptin promoter activity during the differentiation of 3T3-L1 cells. Diabetologia *45*, 140–148.

57. Li, H., Wang, C., He, T., Zhao, T., Chen, Y.Y., Shen, Y.L., Zhang, X., and Wang, L.L. (2019). Mitochondrial Transfer from Bone Marrow Mesenchymal Stem Cells to Motor Neurons in Spinal Cord Injury Rats via Gap Junction. Theranostics *9*, 2017–2035.

58. Nobusue, H., Endo, T., and Kano, K. (2008). Establishment of a preadipocyte cell line derived from mature adipocytes of GFP transgenic mice and formation of adipose tissue. Cell Tissue Res. *332*, 435–446.

59. Ding, M., Liu, C., Shi, R., Yu, M., Zeng, K., Kang, J., Fu, F., and Mi, M. (2020). Mitochondrial fusion promoter restores mitochondrial dynamics balance and ameliorates diabetic cardiomyopathy in an optic atrophy 1-dependent way. Acta Physiol. *229*, e13428.

60. Olichon, A., Baricault, L., Gas, N., Guillou, E., Valette, A., Belenguer, P., and Lenaers, G. (2003). Loss of OPA1 perturbates the mitochondrial inner membrane structure and integrity, leading to cytochrome c release and apoptosis. J. Biol. Chem. *278*, 7743–7746.

OMTM, Volume *31*

Supplemental information

Mitochondrial transplantation reduces lower limb ischemia-reperfusion injury by increasing skeletal muscle energy and adipocyte browning

Jiaqi Zeng, Jianing Liu, Haiya Ni, Ling Zhang, Jun Wang, Yazhou Li, Wentao Jiang, Ziyu Wu, and Min Zhou

Supplementary Information

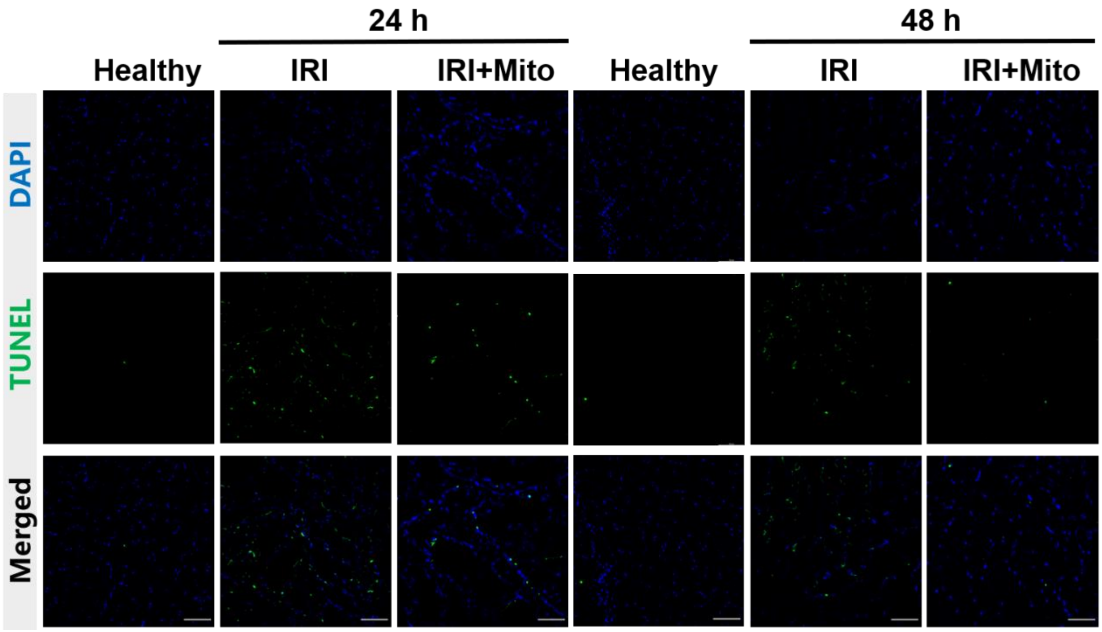

Figure S1. Repair of lower limb IRI by mitochondrial transplantation. Staining of TUNEL-positive cells (blue: nucleus, green: TUNEL, Scale bars: 100 μm).

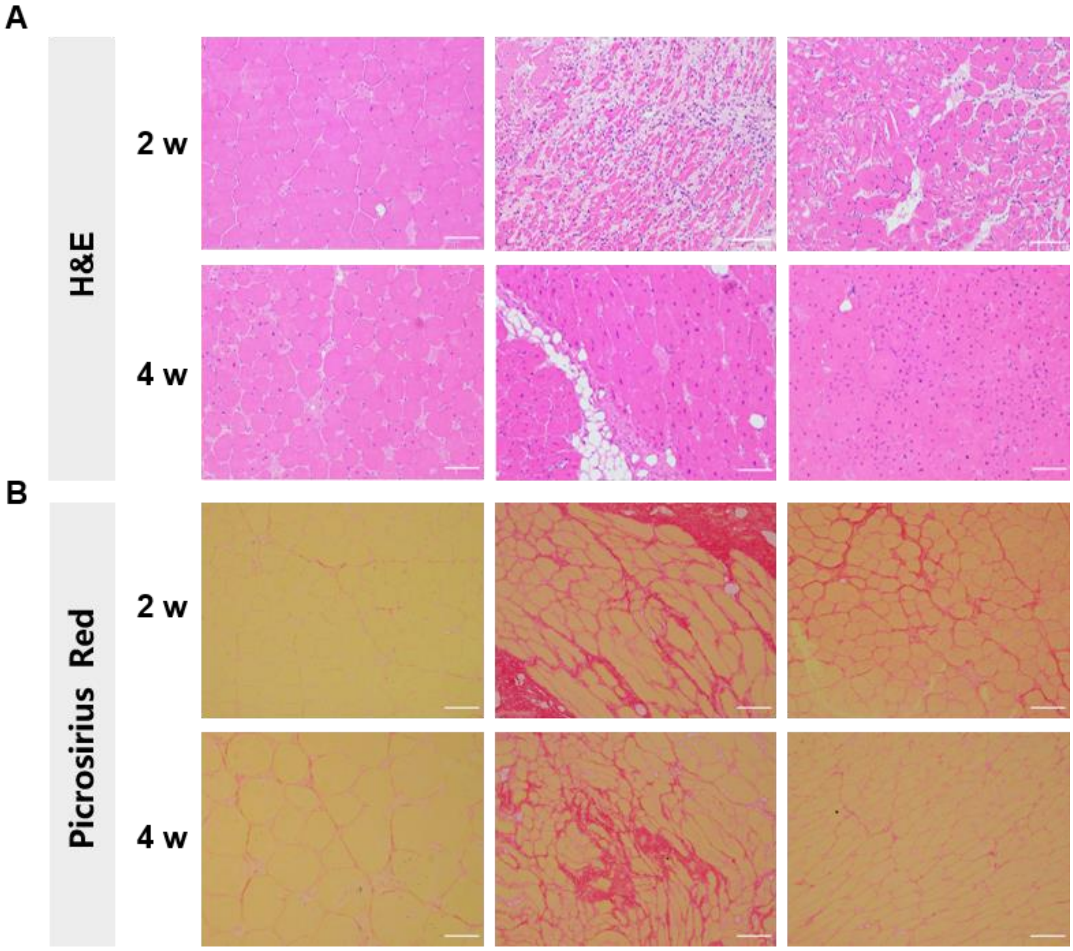

Figure S2. Repair of lower limb IRI by mitochondrial transplantation. (A-B) H&E stain and Picrosirius red stain (Scale bars: 50 μm).

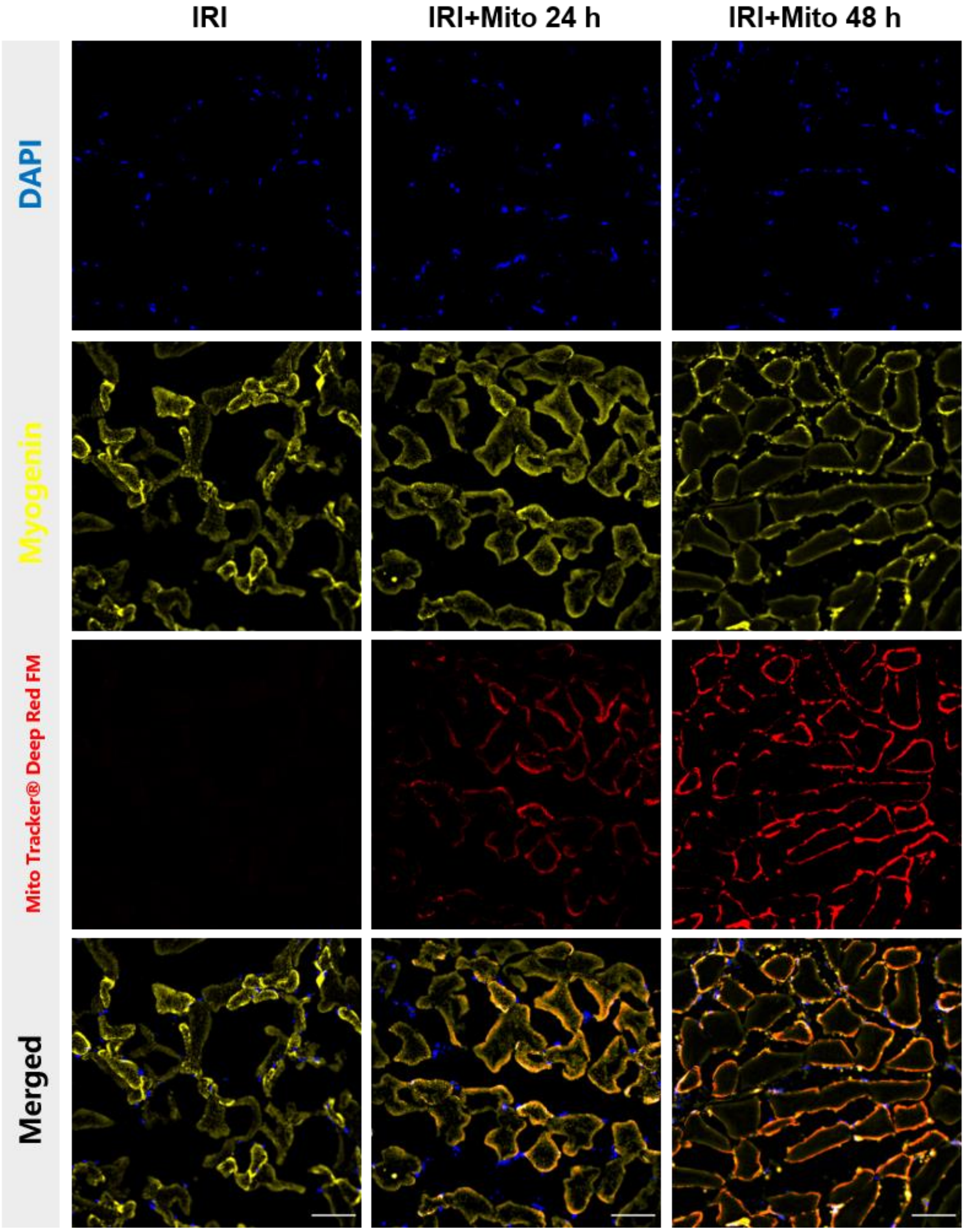

Figure S3. Mitochondrial uptake in *vivo*. CLSM image of hMSCs-derived mitochondria ingestion in mice after 24 h, 48 h of lower limb IRI with mitochondrial transplantation (blue: nucleus, yellow: Myogenin labeled skeletal muscle cells, red: mesenchymal stem cell mitochondria, Scale bars: 50 μm).

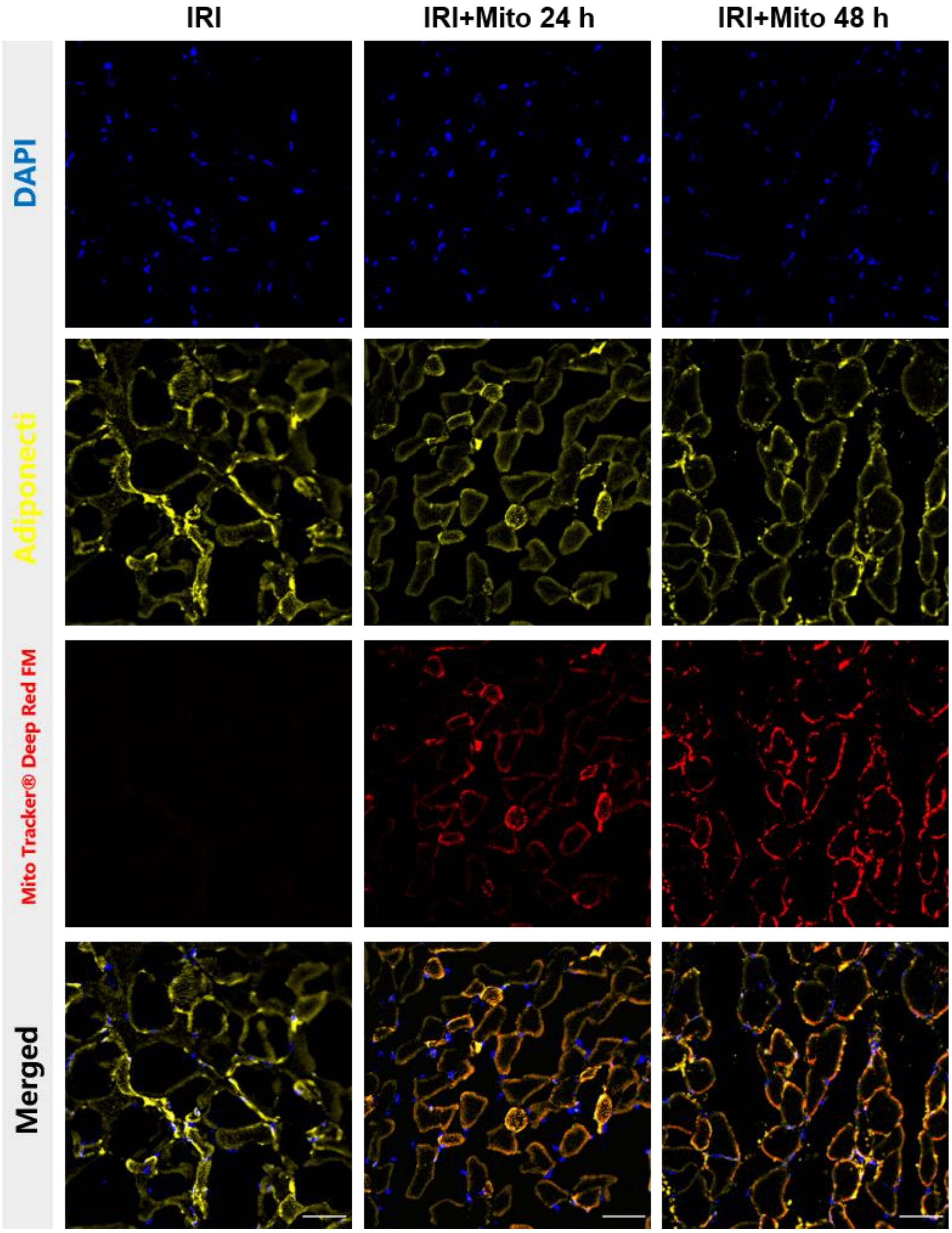

Figure S4. Mitochondrial uptake in *vivo*. CLSM image of hMSCs-derived mitochondria ingestion in mice after 24 h, and 48 h of lower limb IRI with mitochondrial transplantation (blue: nucleus, yellow: adiponectin-labeled adipocytes, red: mesenchymal stem cell mitochondria, Scale bars: 50 μm).

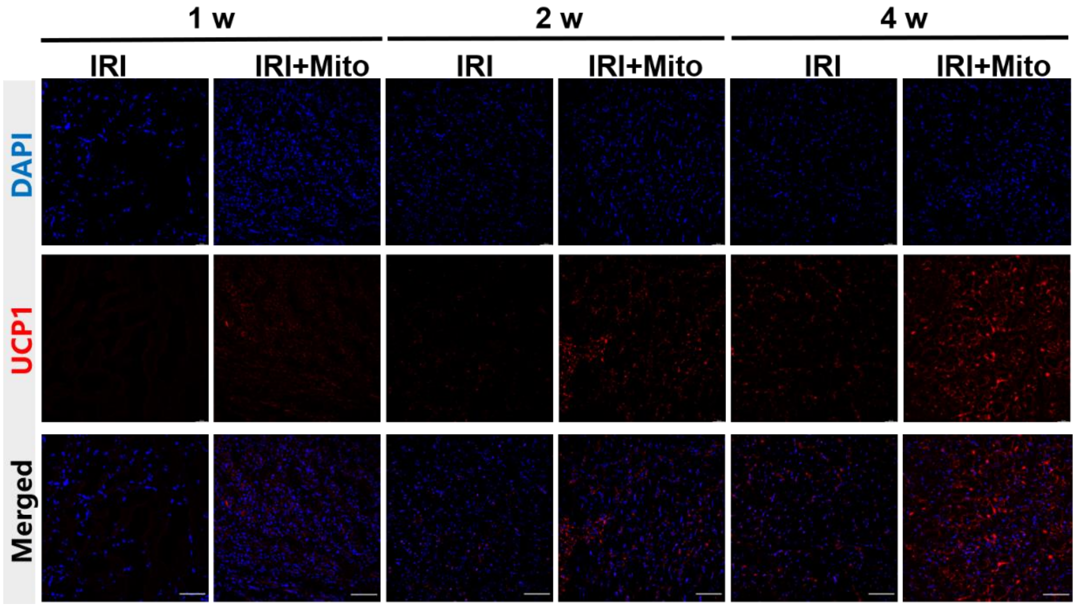

Figure S5. Mitochondrial transplantation promoting effects on the adipocyte browning. Immunofluorescence staining CLSM images was obtained after 1 w, 2 w, and 4 w in the lower limb. (blue: nucleus, red: UCP1, Scale bars: 100 μm)

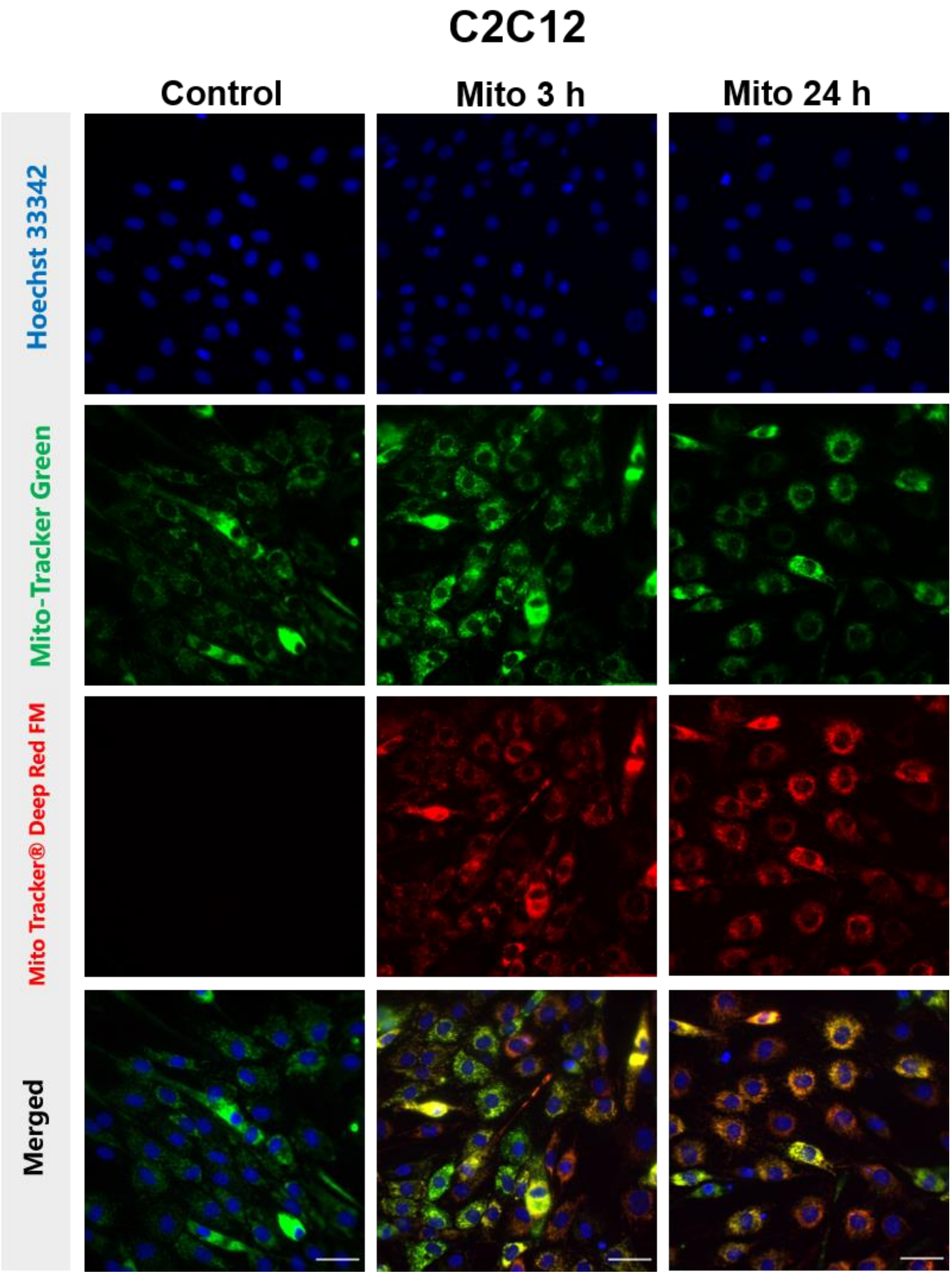

Figure S6. In vitro effects of mitochondrial transplantation on skeletal muscle cells. CLSM images of mitochondrial uptake by C2C12 cells at 3 h and 24 h after hypoxia and reoxygenation. (blue: nucleus, green: original mitochondria of C2C12 cells, red: mitochondria of hMSCs, Scale bars: 50 μm)

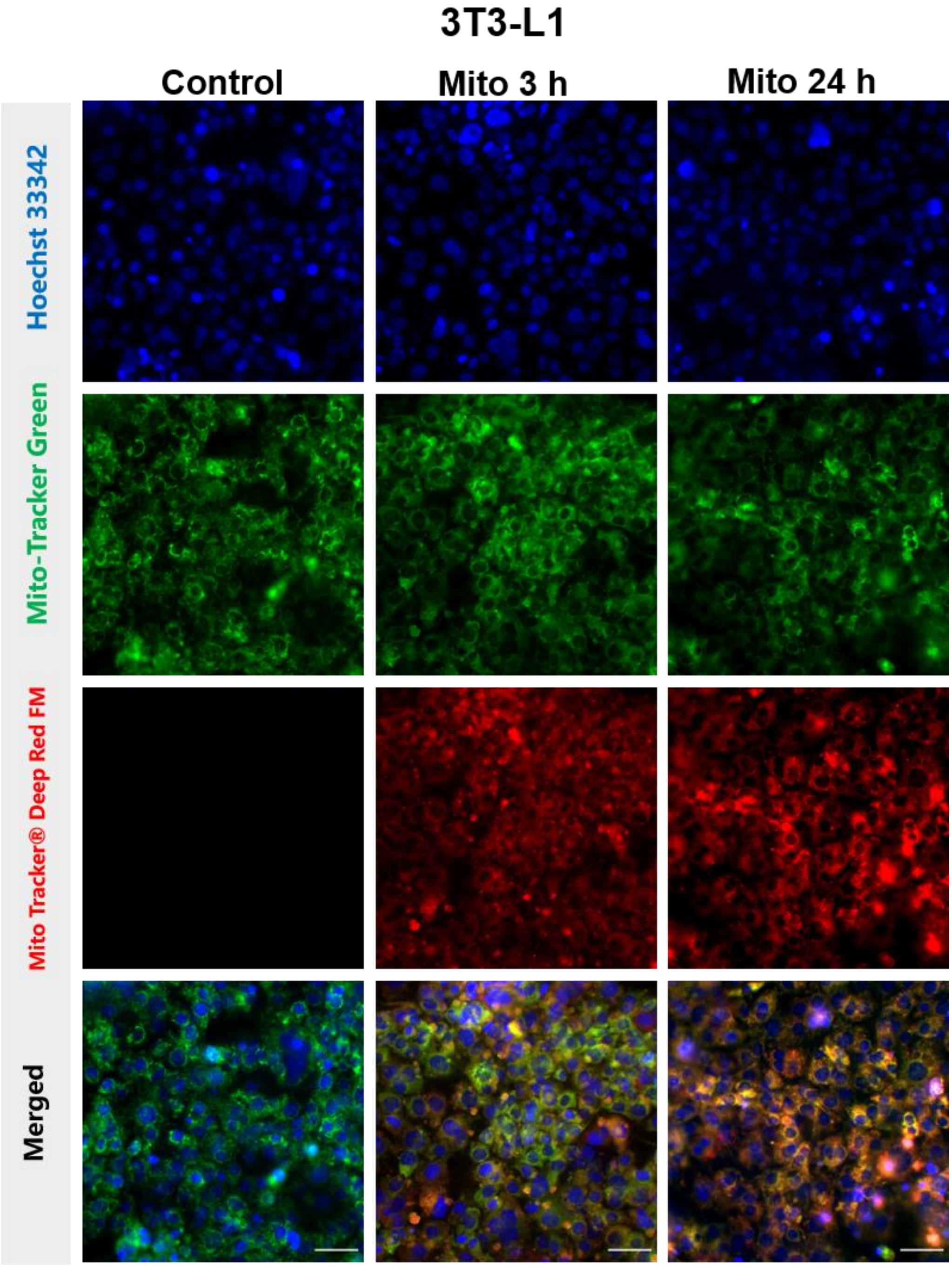

Figure S7. In vitro effects of mitochondrial transplantation on adipocyte browning. CLSM images of mitochondrial uptake by 3T3-L1 cells at 3 h and 24 h. (blue: nucleus, green: 3T3-L1 cell mitochondria, red: hMSCs-derived mitochondria, Scale bars: 50 μm).

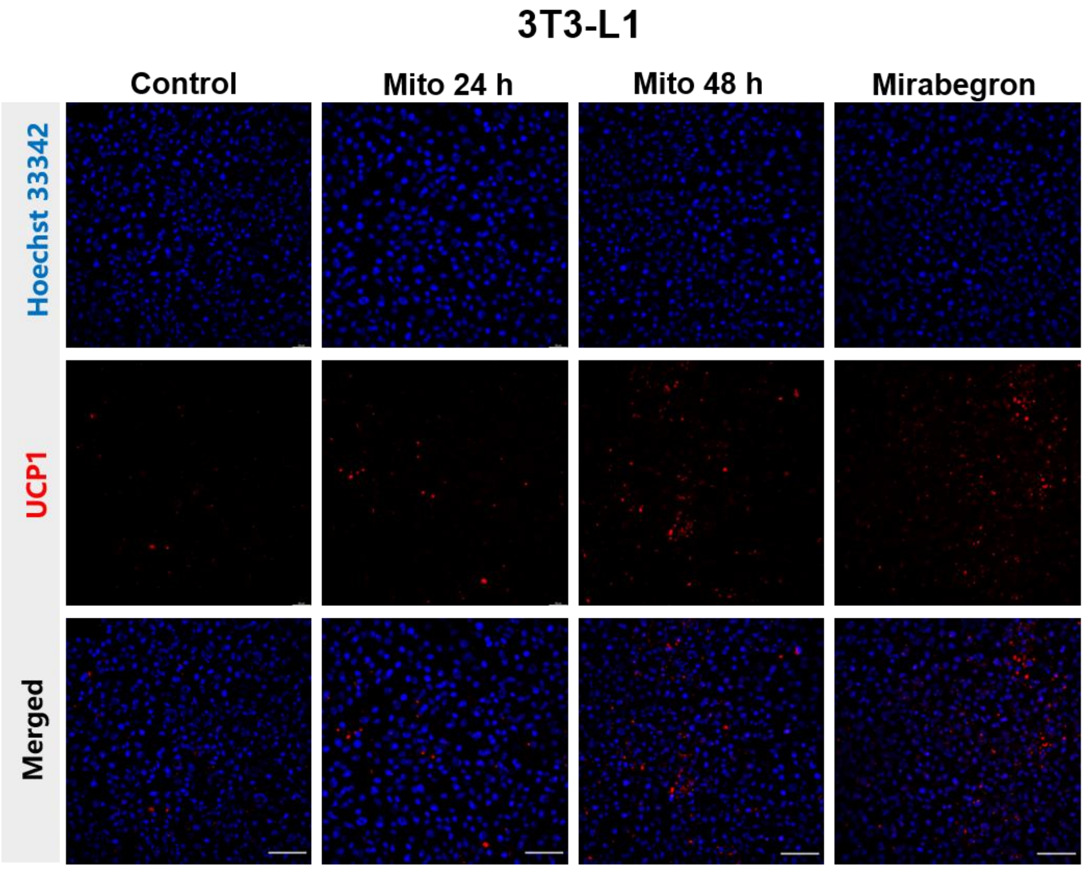

Figure S8. In vitro effects of mitochondrial transplantation on adipocyte browning. CLSM images of UCP1 immunofluorescence. (blue: nucleus, red: UCP1, Scale bars: 100 μm).

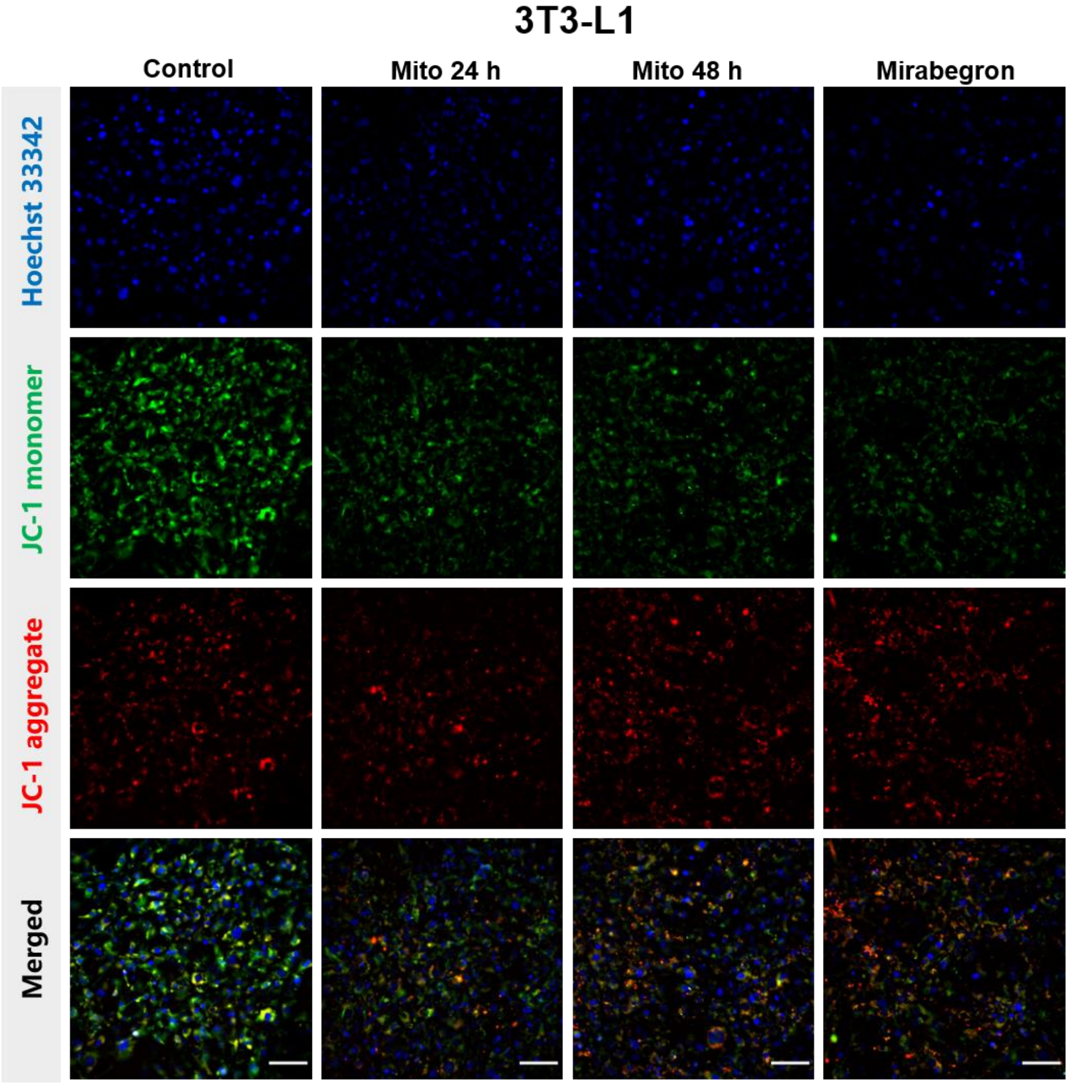

Figure S9. In vitro effects of mitochondrial transplantation on adipocyte browning. JC-1 staining CLSM image (blue: nucleus, green: JC-1 monomer, red: JC-1 aggregate, Scale bars: 100 μm).

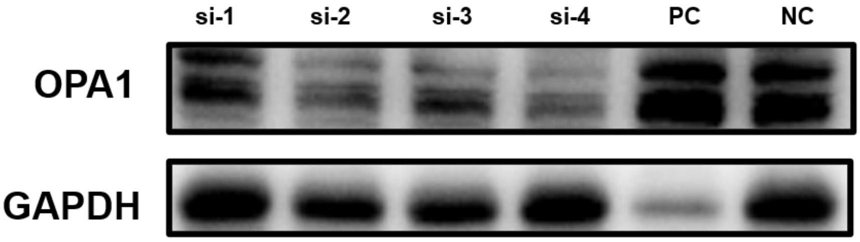

Figure S10. The expression of OPA1 in 3T3-L1-OPA1-siRNA, 3T3-L1-PC-siRNA and 3T3-L1-NC siRNA was compared at the protein level.

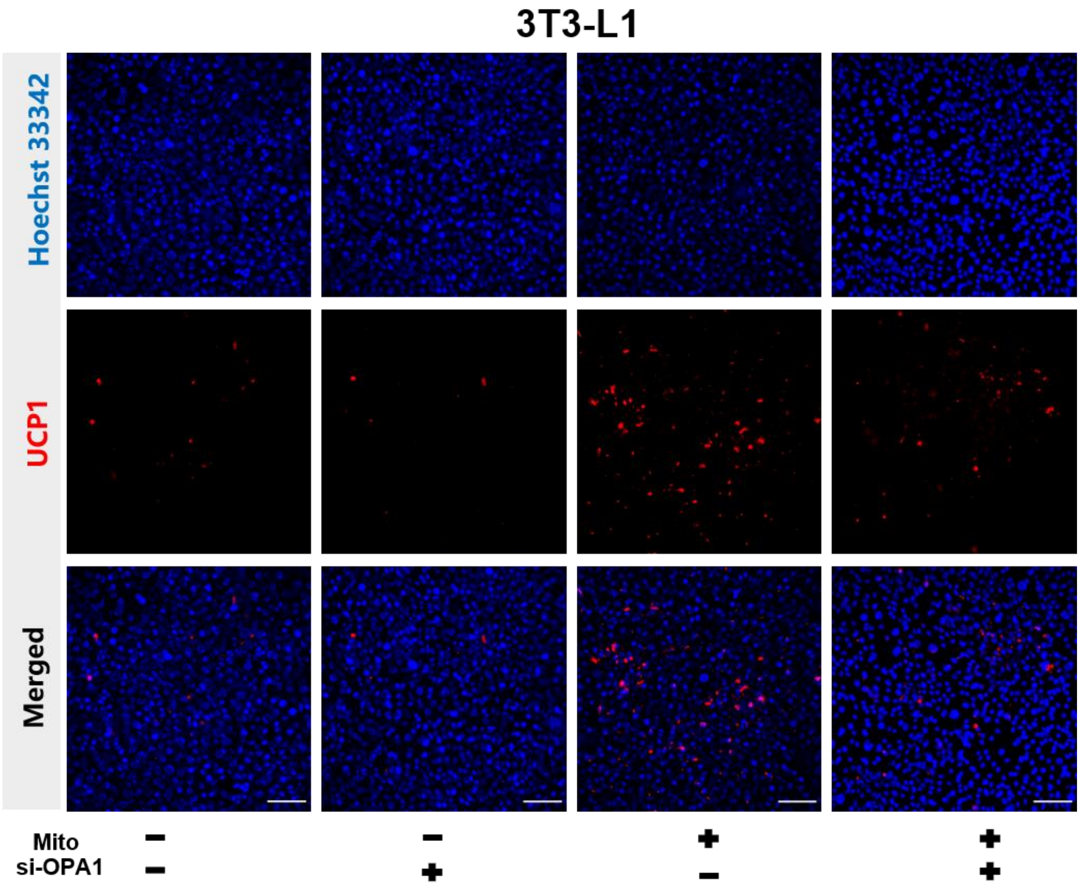

Figure S11. Silencing of OPA1 inhibits adipocyte browning: UCP1 immunofluorescence staining of 3T3-L1 cell blank group, siRNA silencing of OPA1 group, mitochondrial transplantation group, first silencing of OPA1 and then adding mitochondrial group after 48 h of treatment (blue: nucleus, red: UCP1, Scale bars: 100 μm).

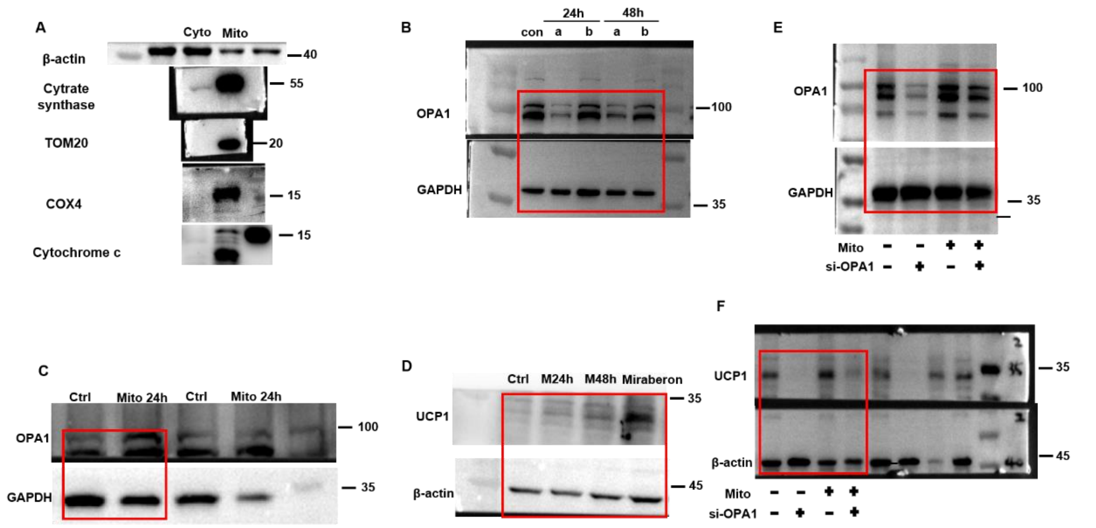

Figure S12. (A) Western blotting analysis of the mitochondria and cytosolic protein markers in the precipitation and supernatant after differential centrifugation. (B) Western blotting of OPA1 in the tissues after 24 h and 48 h (a. lower limb IRI, b. lower limb IRI + Mito). (C) OPA1 protein expression after 24 h of mitochondrial transplantation. (D) Western blotting analysis of UCP1 protein expression. (E-F) Protein levels of OPA1 and UCP1 in 3T3-L1 cell blank group, siRNA silencing OPA1 group, mitochondrial transplantation group, first silencing OPA1 and then adding mitochondria group after 48 h of treatment.

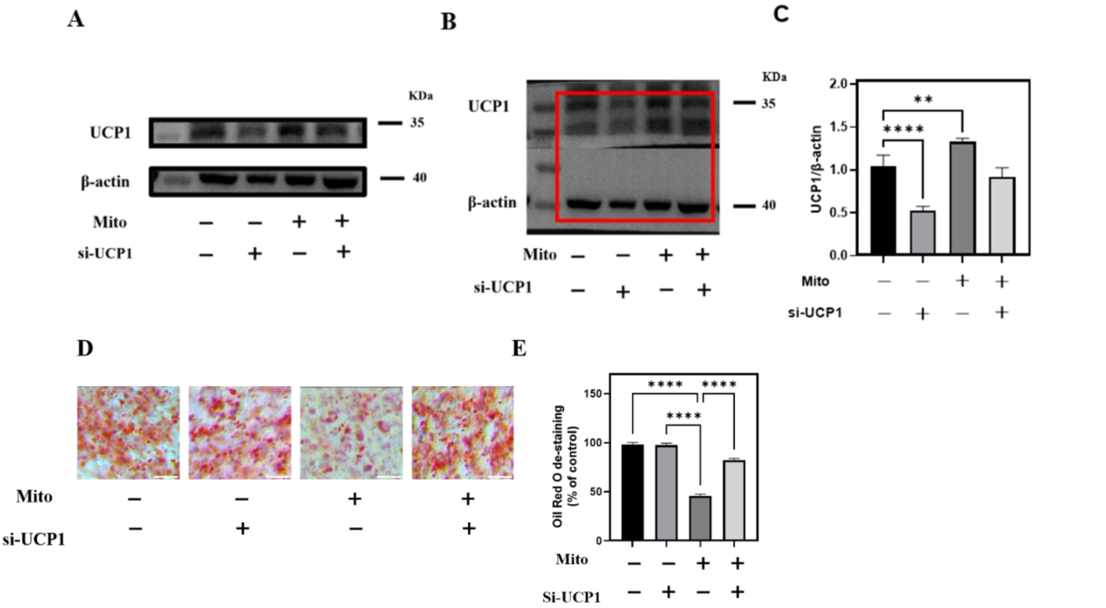

Figure S13. mitochondrial transplantation in the mature adipocytes with knockdown of UCP1. (A-C) The protein expression of UCP1. (D-E) Oil Red O staining and quantification (Scale bar: 2μm). Statistical significance was analyzed using a one-way ANOVA. Experimental data are mean ± s. d. of samples in a representative experiment (n=3). Asterisk (*) denotes statistical significance between bars (**p<0.01, ****

p<0.0001).

Table S1. Results of quantitative analysis of hMSCs mitochondria by Nano-sight

	Stars: Mean+/-Standard Error
**Mean**	337.7+/-23.0 nm
**Mode**	197.3+/-15.4 nm
**SD**	149.4+/-11.2 nm
**D10**	158.4+/-4.4 nm
**D50**	328.6+/-46.3 nm
**D90**	540.9+/-26.4 nm
**Concentration**	5.80e+008+/-1.29e+007particles/ml

Table S2. siRNA2 and siRNA4 (expressed as si-OPA1-2 and si-OPA1-4) sequences used in Western blotting

siRNA name	Forward (5' to 3')	Reverse (5' to 3')
**si-OPA1-2**	CCCGAGACCAUAUCUCUAATT	UUAGAGAUAUGGUCUCGGGTT
**si-OPA1-4**	GUGGCCUUGUUUAAAGAUATT	UAUCUUUAAACAAGGCCACTT

Table S3. si-UCP1 sequences used in Western blotting

siRNA name	Forward (5' to 3')	Reverse (5' to 3')
**si-UCP1**	GCUGUGCGAUGUCCAUGUACATT	UGUACAUGGACAUCGCACAGCTT
